# Toward “CO in a Pill”: Silica-Immobilized Organic CO Prodrugs for Studying the Feasibility of Systemic Delivery of CO via *In Situ* Gastrointestinal CO Release

**DOI:** 10.1021/acs.molpharmaceut.2c01104

**Published:** 2023-02-20

**Authors:** Xiaoxiao Yang, Ravi Tripathi, Minjia Wang, Wen Lu, Abiodun Anifowose, Chalet Tan, Binghe Wang

**Affiliations:** † Department of Chemistry and Center for Diagnostics and Therapeutics, Georgia State University, Atlanta, Georgia 30303, United States; ∇ Department of Pharmaceutics and Drug Delivery, University of Mississippi School of Pharmacy, University, Mississippi 38677, United States; § Department of Pharmaceutical Sciences, University of Tennessee Health Science Center, Memphis, Tennessee 38613, United States

**Keywords:** carbon monoxide, silica gel immobilization, CO prodrug, anti-inflammation, gaseous signaling molecule, oral administration

## Abstract

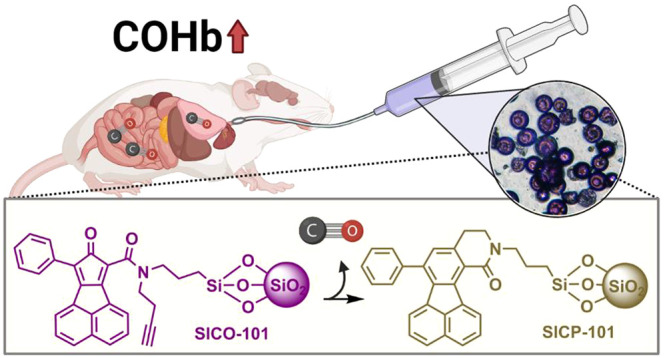

Carbon monoxide (CO), an endogenous signaling molecule, is known to exert a range of pharmacological effects, including anti-inflammation, organ protection, and antimetastasis in various animal models. We have previously shown the ability of organic prodrugs to deliver CO systemically through oral administration. As part of our efforts for the further development of these prodrugs, we are interested in minimizing the potential negative impact of the “carrier” portion of the prodrug. Along this line, we have previously published our work on using benign “carriers” and physically trapping the “carrier” portion in the gastrointestinal (GI) tract. We herein report our feasibility studies on using immobilized organic CO prodrugs for oral CO delivery while minimizing systemic exposure to the prodrug and the “carrier portion.” In doing so, we immobilize a CO prodrug to silica microparticles, which are generally recognized as safe by the US FDA and known to provide large surface areas for loading and water accessibility. The latter point is essential for the hydrophobicity-driven activation of the CO prodrug. Amidation-based conjugation with silica is shown to provide 0.2 mmol/g loading degree, effective prodrug activation in buffer with comparable kinetics as the parent prodrug, and stable tethering to prevent detachment. One representative silica conjugate, **SICO-101**, is shown to exhibit anti-inflammation activity in LPS-challenged RAW264.7 cells and to deliver CO systemically in mice through oral administration and GI CO release. We envision this strategy as a general approach for oral CO delivery to treat systemic and GI-specific inflammatory conditions.

Carbon monoxide (CO) is well-known as a “silent killer” among the general population due to its lethal toxicity at high concentrations. However, the past two decades have witnessed intensive studies in revealing the physiological roles, therapeutic activities, mechanism(s) of action, and innovative delivery approaches of CO.^1−5^ The prospect of developing CO into a therapeutic agent for treating various diseases is on the horizon.^6^ Compared to conventional small-molecule drugs, delivering such a gaseous molecule for therapeutic use poses unique challenges. To deliver CO, inhalation has been used in animal studies and human clinical trials.^6^ However, effective inhalation delivery is highly dependent on individual respiratory functions, requires special hospital equipment to control the dosage, and presents safety issues to patients and healthcare workers.^7^ Furthermore, evidence from animal-model studies shows that nonairway routes, such as gastrointestinal (GI) administration, are expected to have more desirable safety profiles than inhaled CO.^8,9^ The development of nonairway CO-delivery approaches has attracted increasing interest in work on CO in solution,^10^ foam formulation of gaseous CO,^11^ and small-molecule CO donors including metal–carbonyl complexes (termed CO-releasing molecules or CORMs),^2,9^ photoactivated metal-^12,13^ and organic-based^14−19^ CO donors, and organic CO prodrugs capable of CO release under physiological conditions,^20−23^ which allows dosing CO through oral or injection administration. Meinel and colleagues developed a binary microreactor in tablet form that could be a general formulation for certain types of CO delivery.^24,25^ For example, CORM-2 is a Ru(II)-carbonyl complex and has been found to possess issues of incertitude in CO release^26^ and CO-independent reactivity and activities.^26−28^ However, when CORM-2 was coated with sodium dithionate in this binary microreactor, the incorporation of this “triggering” agent into the tablet led to improved consistency in CO release.^24^

Among the organic CO prodrugs we have developed, the most studied class relies on cheletropic extrusion of carbon monoxide from a norbornadiene-7-one moiety for CO release^20,22,29−33^ under physiological conditions. These prodrugs have demonstrated efficacy in animal models of kidney injury, gastric injury, liver injury, and general inflammation. As is true with any prodrug strategy, there is the need to minimize the unintended effects from the “carrier” portion of a prodrug. We desire the same in the further development of CO prodrugs. Along this line, we have worked on preparing prodrugs using benign carriers such as artificial sweeteners saccharine and acesulfame^34^ and innovative formulations, which allowed for physical trapping of the “carrier” portion in a solid-phase matrix such as activated charcoal.^35^ In this study, we focus on assessing the feasibility of a third approach by immobilizing an organic CO prodrug to a solid-phase matrix with the hope that (1) the solid-phase matrix is known to be benign and is not absorbed into the systemic circulation; (2) after immobilization, the CO extrusion reaction still occurs at a comparable rate as the parent prodrug in solution; (3) the conjugation chemistry offers the kind of stability so that the “carrier” portion remains tethered after CO release; and (4) the immobilized prodrug offers comparable CO bioavailability, as measured by the carboxyhemoglobin (COHb) level, as the prodrug itself.

To address the first issue, we considered various options and settled on silica gel for the feasibility studies. Amorphous silica gel is a biocompatible and generally benign material that has been widely used as a drug excipient and food additive.^36^ Depending on the size, there are mainly two types of silica for use as drug delivery vehicles for dosing via various routes: silica nanoparticles and microparticles (in the μm size range). Silica nanoparticles are known to be cell permeable^37,38^ and are not desirable for the intended applications. On the other hand, silica microparticles, either synthesized or obtained from natural biosilica such as silica diatoms, have been studied in encapsulating probiotic bacteria or drugs for oral delivery.^37^ Silica microparticle encapsulation offers gastro-retention and gastro-resistance, allowing for local action in the GI tract and shielding the loaded drug from the severe gastric environment or, conversely, protecting the gastric mucus against drug irritation.^37^ As for our applications, silica gel offers thermal stability, chemical inertness, biocompatibility, and large water-accessible surface areas,^39^ which are critical for the hydrophobicity-driven activation of our Diels–Alder reaction-based CO prodrugs (Figure 1).^32^ For additional considerations, we chose to use spherical silica gel instead of irregular silica gel to facilitate oral administration via gavage and to minimize potential irritation. Further, we chose the particle size of 20–45 μm to allow for easy passage through a 20- or 18-gauge gavage needle as a suspension in 3% carboxymethylcellulose (CMC). An additional consideration is to prevent the absorption of the particles and consequently the immobilized compounds by staying far above the 5 μm particle size, which is reported to be the threshold for absorption by the small intestine.^40^

**Figure 1:**
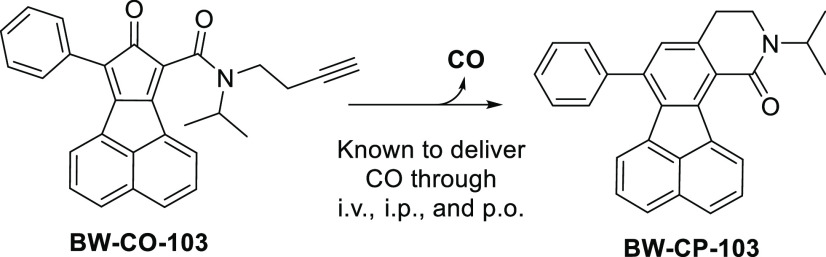
An organic CO prodrug that depends on hydrophobicity-driven Diels–Alder reaction for activation.

CO prodrug **BW-CO-103**(32) (Figure 1) was selected as the CO donor for immobilization to silica gel, owing to its modest CO release kinetics (half-life of about 1.2 h)^41^ and available chemistry for tethering to silica gel. As illustrated in Scheme 1, bromobutyne was substituted with aminopropyl triethoxysilane (APTES) or aminopropyl diisopropylsilane (APDIPS) to introduce a linker. Two types of silane linkers were used to evaluate the influence of conjugation chemistry on CO loading degree. In addition, due to the instability of the triethyloxysilane moiety to moisture during purification, we could not separate the amine **1a** with flash column chromatography. On the other hand, with diisopropyl substitution to shield the silanol group,^42^**1b** was more stable and easier to separate by column chromatography. Due to the spontaneous CO release of the alkyne-derived cyclopentadienone through intramolecular Diels–Alder reaction, the formation of such “armed” CO-releasing moiety was designed as the last step. As such, the diketone intermediate **3** was synthesized and loaded on silica gel by heating in toluene according to a literature procedure.^43^ After loading, intermediates **4a** and **4b** were reacted with acenaphthoquinone through an aldol condensation reaction to get the “armed” immobilized CO prodrugs **SICO-101** and **-102** as purple solid (Figure 2A, Figure S1). To assess the relationship between surface area and CO loading degree, two types of silica gels with different porosity and thus different surface areas (Table 1) were used to conjugate with intermediate **3b** to form **SICO-102-I** and **SICO-102-II**. Immobilization of the CO prodrug was confirmed with solid-phase magic-angle NMR (MAS NMR), which showed aromatic and aliphatic proton and carbon signals (Figure S2).

**Scheme 1:**
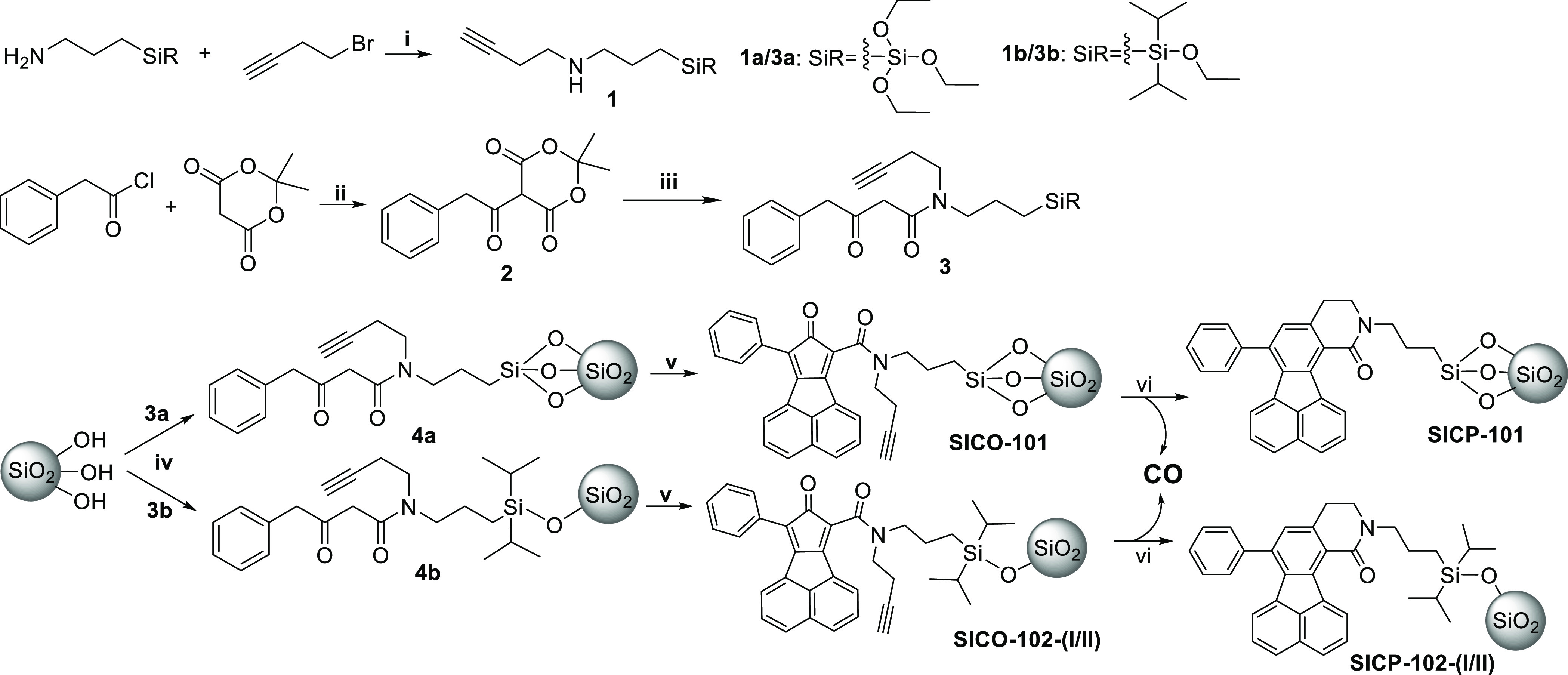
Synthesis of Silica-Immobilized CO Prodrugs *Reagents and conditions*: (**i**) ACN, reflux; (**ii**) pyridine, DCM, 0 °C–rt; (**iii**) compound **1**, TMS-Cl, toluene, 90 °C; (**iv**) compound **3**, toluene, 90 °C heating in sealed tube for 36 h; (**v**) acenaphthoquinone, TEA, DMF, 45 °C, 1.5 h; then Ac_2_O, H_2_SO_4_, 0 °C, 30 min; (**vi**) DI water or buffer, 37–65 °C, 12 h.

**Figure 2:**
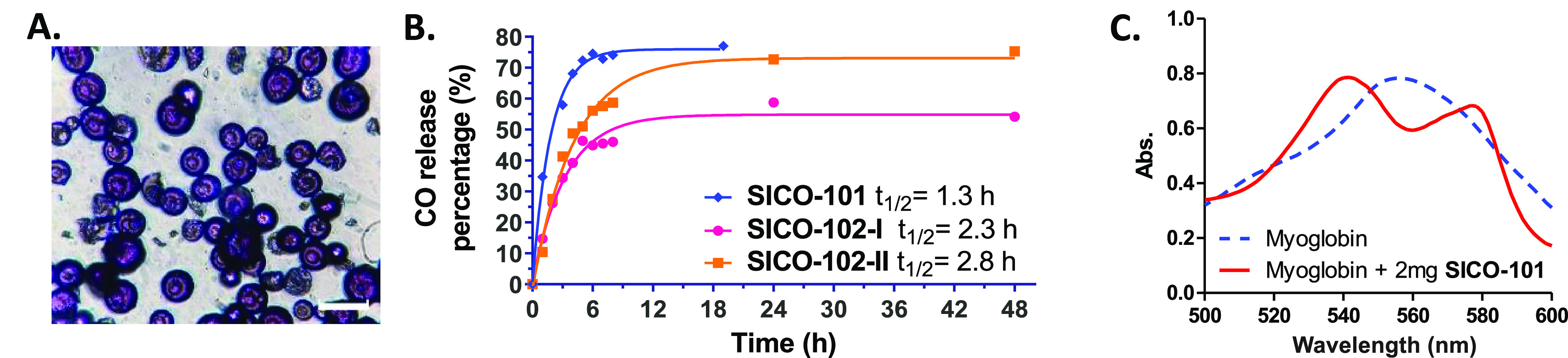
(A) Microscopic morphology of **SICO-101** (scale bar: 50 μm); (B) CO release kinetics of **SICO**s (nonlinear regression of the data is shown as the solid trace); (C) myoglobin assay showing characteristic Q-band absorption of carboxymyoglobin after incubation with **SICO-101** at 37 °C for 1 h.

**Table 1:** CO Release Profiles of Silica Gel-Immobilized CO Prodrugs

	silica gel specification					
compound ID	size (μm)	porosity (Å)	surface area (m^2^/kg)	loading degree of CO (mmol/g)^a^	max. CO release % at 37 °C	CO release *t*_1/2_ at 37 °C (h)
**SICO-101**	20–45	70	450–550	0.17 (0.13)	77%	1.3
**SICO-102-I**	20–45	70	450–550	0.20 (0.10)	54%	2.3
**SICO-102-II**	20–45	100	270	0.08 (0.06)	75%	2.8

a Results in the parentheses denote the bona fide CO loading degree achieved at 37 °C.

The immobilized prodrugs were first assessed for their loading degree and CO release. It is well-defined that CO release from the CO prodrugs through intramolecular Diels–Alder reaction is facilitated by hydrophobic force in an aqueous environment^32^ and by increasing reaction temperature. Further, solvent (water) infiltration into silica gel can also be driven by increasing the temperature.^44^ Therefore, to achieve complete CO release for loading degree determination, **SICO-101** and **SICO-102** were incubated at 65 °C overnight in PBS in a headspace vial (Table 1). Among the three **SICO**s, **SICO-102-I** showed the highest overall CO loading degree of 0.2 mmol/g; as expected, **SICO-102-II** with less surface area also resulted in a much-decreased CO loading degree, indicating an expected correlation between surface area and CO loading degree. However, when evaluating the CO release profile of these **SICOs** under physiological conditions (37 °C), **SICO-102-I** was found to release a maximum of only 54% of the overall CO load, resulting in a bona fide CO loading degree of 0.1 mmol/g. On the other hand, **SICO-101** released 77% of the loaded CO within 6 h, giving a slightly higher bona fide CO loading degree (0.13 mmol/g) under near-physiological conditions. Presumably, compared to APTES, APDIPS may increase the hydrophobicity of the loaded silica gel, rendering it less water-accessible^42^ for prodrug activation, especially for the CO prodrug buried inside the silica cavity. Studies of CO-release kinetics (Figure 2B) showed that immobilization through an APTES linker led to CO-release kinetics (*t*_1/2_ of about 1.3 h) comparable to that of the parental CO prodrug **BW-CO-103**, indicating the water-accessible nature of the conjugated prodrug. Such properties meet our expectation of maintaining similar CO release kinetics after immobilization. Since **BW-CO-103** suffers from low water solubility and requires a cosolvent such as DMSO or a solubilizer such as Kolliphor HS15 for solubilization for *in vitro*(32) and *in vivo* studies,^45^ silica gel immobilization allows for resolution of this solubility issue and yet provides water-accessible surface features to facilitate CO release due to the hydrophilic surface silanol groups.^46^ Interestingly, **SICO-102** with an APDIPS linker led to a slower CO release compared to the parent CO prodrug **BW-CO-103**, with a half-life of about 2 h in general, presumably due to the increased hydrophobicity of the diisopropyl-decorated silica surface.^42^ CO release from **SICO-101** was also verified by a widely used myoglobin assay^4^ (Figure 2C). It should be noted that since only CO is released to the myoglobin assay solution and the chromogenic CO prodrug posed no interference to the spectroscopic measurements, such an assay was done by directly incubating **SICO-101** with a myoglobin solution without the need for a two-compartment assay as we did for **BW-CO-103**.^32^ In addition, a preliminary shelf life evaluation showed **SICO-101** being stable at −20 °C for a month without a significant decrease in CO release yield (Table S2). However, prolonged storage at room temperature or elevated temperature did compromise the CO release yield and should be avoided.

With confirmed CO release yield and kinetics in a biologically relevant medium, **SICO-101** was chosen for further biological evaluation. CO is well-known to exert anti-inflammatory activity both *in vitro* and *in vivo*.^47^ Liposaccharide (LPS)-induced release of TNF-α from RAW264.7 cells has been extensively used as an *in vitro* model to evaluate the anti-inflammatory activity of CO prodrugs and CO gas.^32,34^ Therefore, we sought to test the anti-inflammatory activity of **SICO-101** and the corresponding CO-released byproduct **SICP-101** by an ELISA assay (Figure 3). To avoid potential interference by silica gel particles, we physically separated the RAW264.7 cells from the silica gel in the culturing medium by using Transwell cell culture inserts, as CO released in the culture medium can readily diffuse to the cells in the upper compartment. In this two-chambered system, CO is released into the cell-culture medium and offers an enhanced level of diffusion and exchange with the surrounding air. To mitigate this, we used a higher dose (about 2.1 mM in total) than what we normally use for a nonimmobilized prodrug. It should be noted that the total CO prodrug concentration does not represent the available CO concentration at a given moment, as we have demonstrated in other studies.^48^ As shown in Figure 3, in the chronic LPS-challenge assay, LPS significantly induced TNF-α expression in the RAW264.7 cell culture after 12 h. Co-incubation with 4–16 mg/mL **SICO-101** significantly reduced TNF-α expression levels, indicating the anti-inflammatory effects of CO generated by **SICO-101**. Meanwhile, cell viability was not significantly affected by **SICO-101** or **SICP-101** (Figure S3).

**Figure 3:**
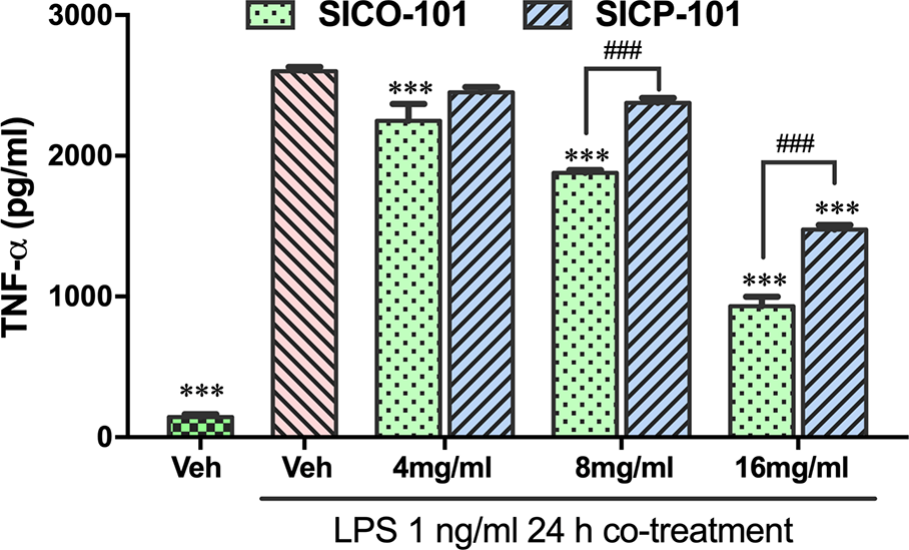
TNF-α levels in the RAW264.7 cell cultures treated with **SICO-101** and **SICP-101**. Cells were coincubated with 1 ng/mL LPS and **SICO-101** or **SICP-101** for 24 h. Cells were seeded in Transwell in the upper compartment, and **SICO-101** and **SICP-101** were added to the culture medium in the lower compartment. Mean ± SD, ** *p* < 0.01 and *** *p* < 0.001 vs LPS-only group; # *p* < 0.05 and ### *p* < 0.001 vs corresponding **SICP** group; culture medium is the vehicle group (*n* = 3).

The goal of developing such a silica gel-immobilized CO prodrug is to enable oral delivery of CO to achieve effective systemic availability. Such an administration route would require **SICO-101** to release CO under the acidic conditions of the gastric fluid. By using a simulated gastric fluid (SGF, pH 1.2), CO release was confirmed with a slight decrease in half-life, and a bona fide CO-release yield (76%) in the SGF (Figure 4A) similar to that in PBS. Such fast CO release under acidic conditions was also seen with another Diels–Alder reaction-based CO prodrug, **BW-CO-111**.^49^ Moreover, since the byproduct after CO release is fluorescent, it allows for easy quantification of the potential detachment of the CO prodrug and the byproduct after CO release. Fluorescence spectrophotometer analysis of the SGF, SIF, and PBS after CO release from **SICO-101** did not detect a meaningful quantity (less than 0.1%) of the fluorescent release product (Figure S4 and Table S1), indicating the stable nature of immobilization for both the CO prodrug and CO-released byproduct.

**Figure 4:**
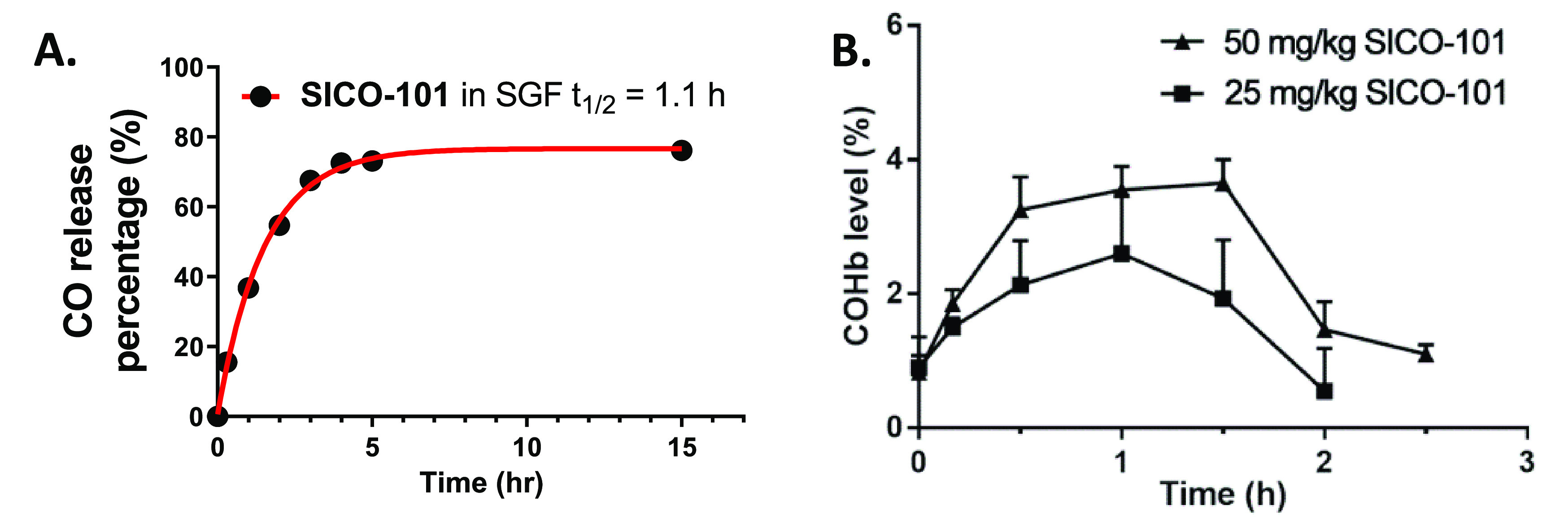
*In vitro* CO release and *in vivo* pharmacokinetic profiles of **SICO-101**. (A) CO release kinetics of **SICO-101** in SGF by using GC (solid trace shows the nonlinear regression of the data); (B) *in vivo* COHb profiles in mice after oral administration of **SICO-101** (mean + SD, *n* = 3).

Lastly, we verified the ability of **SICO-101** to deliver systemically available CO through oral administration. **SICO-101** was mixed with 3% CMC aqueous solution as a slurry that can readily pass through the 18-gauge gavage needle. This CMC formulation was given to mice through oral gavage, followed by drawing peripheral blood to test the COHb levels at different time points. To compare the CO prodrug with and without immobilization, we chose to use a dose of **SICO-101** on par with that of the native CO prodrug **BW-CO-103** (25 and 50 mg/kg). As shown in Figure 4B and Table 2, **SICO-101** dose-dependently increased mouse blood COHb levels. The peak COHb levels of **SICO-101** given at a dose equivalent to 50 mg/kg and 25 mg/kg of the prodrug (calculated based on 0.17 mmol/g substitution degree, 350 mg of the silica conjugate is equivalent of 25 mg of the prodrug) were about 4.3% and 2.4%, respectively. As the bona fide CO release yield is about 77% for **SICO-101**, the COHb AUC achieved by **SICO-101** is on par with that of **BW-CO-103** at the same CO prodrug dose (25 mg/kg) after calibrating against the CO loading degree and effective release yield. Specifically, a 25 mg/kg dose of **BW-CO-103** gave rise to about COHb AUC of 4.1 ± 0.3%·h, whereas the equivalent in the form of **SICO-101** gave a COHb AUC of 2.4 ± 0.5%·h, which is about 59% of the CO bioavailability dosed by **BW-CO-103**. Considering individual differences among mice and experimental variations, such coherence confirms the delivery efficiency of **SICO-101** as being comparable to that of **BW-CO-103**. Previously, we have shown that oral administration of 25 mg/kg **BW-CO-103** in mice resulted in systemic exposure of the CO-released byproduct **BW-CP-103** with an AUC of 7.7 ± 0.6 μM·h and bioavailability of about 25% compared to 5 mg/kg i.v. administration.^45^ As the silica immobilization approach has been shown to retain the byproduct on the silica, the adsorption through the GI tract and systemic exposure are mostly circumvented. It should be noted that throughout the animal study procedure after oral administration of the **SICO-101** formulation, there was no noticeable change in the appearance or behavior of the mice, indicating minimum acute safety concerns for this formulation. The bulk (if not all) of **SICO-101** after releasing CO is expected to be excreted in feces because of the large size of the silica particles. Nevertheless, the biosafety of the formulation needs further evaluation for the future development of this approach.

**Table 2:** CO Delivery Efficiency of **SICO-101** and **BW-CO-103** (*n* = 3)

compound ID	route of administration	CO prodrug dose (as in **BW-CO-103**, mg/kg)	silica gel dose (mg/kg)	COHb AUC (%·h)	CO delivery efficiency^a^ (%)
SICO-101	*p.o.*	50	700	4.3 ± 0.3	6.1
	*p.o.*	25	350	2.4 ± 0.5	6.9
**BW-CO-103** (via Kolliphor)	*i.v*.	5	N.A.	7.0 ± 0.9	100
	*p.o.*	25	N.A.	4.1 ± 0.3	11.7

a Calculated as 100% × (AUC_COHb_ /p.o. Dose_COprodrug_)/(7.0/5)_(i.v.AUC/i.v.Dose)_. N.A.: Not available.

## Conclusion

To summarize, we have developed a third approach for oral CO delivery to the systemic circulation using a CO prodrug while minimizing the unintended effects of the “carrier” moiety. In this case, a CO prodrug is immobilized to spherical silica gels. The resulting immobilized prodrug features stable attachment of the CO prodrug, spontaneous CO release in the biological environment, and retention of the stable conjugation after CO release. The CO loading degree and the CO release kinetics of the silica-immobilized CO prodrugs are dependent on the surface area and the linker chemistry. A representative candidate, **SICO-101**, was found to exert anti-inflammatory activity in the LPS-challenged model in RAW264.7 cells. We also showed **SICO-101** was able to deliver CO to mice through oral administration with kinetic and bioavailability parameters comparable to that of the parent prodrug **BW-CO-103**. We have met all four criteria that we set for this approach: using a benign matrix, ensuring water accessibility to the immobilized prodrug for CO-release kinetics comparable to that the parent prodrug, stable conjugation chemistry to allow the “carrier” portion to remain tethered after CO release, and achieving CO bioavailability comparable to that of the prodrug itself. To the best of our knowledge, there has not been a study of delivering CO through oral administration of immobilized CO prodrugs. With further optimization for high CO loading degree and CO release kinetics, the strategy described may generate potential candidates for CO delivery to treat various inflammatory diseases, including in the gastrointestinal system.

## Supporting Information Available

Supplemental figures, materials and methods, chemical synthesis and characterization of **SICOs**, and NMR spectra (PDF)

## References

[ref1] Wu L.; Wang R. Carbon monoxide: endogenous production, physiological functions, and pharmacological applications. Pharmacol Rev. 2005, 57, 585–630. 10.1124/pr.57.4.3. 16382109

[ref2] Motterlini R.; Otterbein L. E. The therapeutic potential of carbon monoxide. Nat. Rev. Drug Discovery 2010, 9, 728–743. 10.1038/nrd3228. 20811383

[ref3] Romao C. C.; Blattler W. A.; Seixas J. D.; Bernardes G. J. Developing drug molecules for therapy with carbon monoxide. Chem. Soc. Rev. 2012, 41, 3571–83. 10.1039/c2cs15317c. 22349541

[ref4] Heinemann S. H.; Hoshi T.; Westerhausen M.; Schiller A. Carbon monoxide--physiology, detection and controlled release. Chem. Commun. 2014, 50, 3644–60. 10.1039/C3CC49196J. PMC4072318 24556640

[ref5] Yuan Z.; De La Cruz L. K.; Yang X.; Wang B. Carbon Monoxide Signaling: Examining Its Engagement with Various Molecular Targets in the Context of Binding Affinity, Concentration, and Biologic Response. Pharmacol Rev. 2022, 74, 825–875. 10.1124/pharmrev.121.000564. PMC9553107 35738683

[ref6] Wang B.; Otterbein L. E. Carbon Monoxide in Drug Discovery: Basics, Pharmacology, and Therapeutic Potential, 1st ed.; John Wiley and Sons: Hoboken, NJ, 2022.

[ref7] Tripathi R.; Yang X.; Ryter S. W.; Wang B. Carbon Monoxide as a Therapeutic for Airway Diseases: Contrast and Comparison of Various CO Delivery Modalities. Curr. Top Med. Chem. 2021, 21, 2890–2908. 10.2174/1568026621666211116090917. PMC9206592 34784868

[ref8] Yang X.; Lu W.; Wang M.; Tan C.; Wang B. ″CO in a pill″: Towards oral delivery of carbon monoxide for therapeutic applications. J. Controlled Release 2021, 338, 593–609. 10.1016/j.jconrel.2021.08.059. PMC8526413 34481027

[ref9] Steiger C.; Hermann C.; Meinel L. Localized delivery of carbon monoxide. Eur. J. Pharm. Biopharm. 2017, 118, 3–12. 10.1016/j.ejpb.2016.11.002. 27836646

[ref10] Belcher J. D.; Gomperts E.; Nguyen J.; Chen C.; Abdulla F.; Kiser Z. M.; Gallo D.; Levy H.; Otterbein L. E.; Vercellotti G. M. Oral carbon monoxide therapy in murine sickle cell disease: Beneficial effects on vaso-occlusion, inflammation and anemia. PLoS One. 2018, 13, e0205194 10.1371/journal.pone.0205194. PMC6181332 30308028

[ref11] Byrne J. D.; Gallo D.; Boyce H.; Becker S. L.; Kezar K. M.; Cotoia A. T.; Feig V. R.; Lopes A.; Csizmadia E.; Longhi M. S.; Lee J. S.; Kim H.; Wentworth A. J.; Shankar S.; Lee G. R.; Bi J.; Witt E.; Ishida K.; Hayward A.; Kuosmanen J. L. P.; Jenkins J.; Wainer J.; Aragon A.; Wong K.; Steiger C.; Jeck W. R.; Bosch D. E.; Coleman M. C.; Spitz D. R.; Tift M.; Langer R.; Otterbein L. E.; Traverso G. Delivery of therapeutic carbon monoxide by gas-entrapping materials. Sci. Transl Med. 2022, 14, eabl4135 10.1126/scitranslmed.abl4135. PMC9576196 35767653

[ref12] Jiang Q.; Xia Y.; Barrett J.; Mikhailovsky A.; Wu G.; Wang D.; Shi P.; Ford P. C. Near-Infrared and Visible Photoactivation to Uncage Carbon Monoxide from an Aqueous-Soluble PhotoCORM. Inorg. Chem. 2019, 58, 11066–11075. 10.1021/acs.inorgchem.9b01581. 31369245

[ref13] Kawahara B.; Gao L.; Cohn W.; Whitelegge J. P.; Sen S.; Janzen C.; Mascharak P. K. Diminished viability of human ovarian cancer cells by antigen-specific delivery of carbon monoxide with a family of photoactivatable antibody-photoCORM conjugates. Chem. Sci. 2020, 11, 467–473. 10.1039/C9SC03166A. PMC7067254 32190266

[ref14] Lazarus L. S.; Dederich C. T.; Anderson S. N.; Benninghoff A. D.; Berreau L. M. Flavonol-Based Carbon Monoxide Delivery Molecule with Endoplasmic Reticulum, Mitochondria, And Lysosome Localization. ACS Med. Chem. Lett. 2022, 13, 236–242. 10.1021/acsmedchemlett.1c00595. PMC8842101 35178180

[ref15] Weinstain R.; Slanina T.; Kand D.; Klán P. Visible-to-NIR-Light Activated Release: From Small Molecules to Nanomaterials. Chem. Rev. 2020, 120, 13135–13272. 10.1021/acs.chemrev.0c00663. PMC7833475 33125209

[ref16] Abeyrathna N.; Washington K.; Bashur C.; Liao Y. Nonmetallic carbon monoxide releasing molecules (CORMs). Org. Biomol Chem. 2017, 15, 8692–8699. 10.1039/C7OB01674C. 28948260

[ref17] Stackova L.; Russo M.; Muchova L.; Orel V.; Vitek L.; Stacko P.; Klan P. Cyanine-Flavonol Hybrids for Near-Infrared Light-Activated Delivery of Carbon Monoxide. Chem. Eur. J. . 2020, 26, 13184–13190. 10.1002/chem.202003272. PMC7693251 32885885

[ref18] Sutton D. A.; Popik V. V. Sequential Photochemistry of Dibenzo[a,e]dicyclopropa[c,g][8]annulene-1,6-dione: Selective Formation of Didehydrodibenzo[a,e][8]annulenes with Ultrafast SPAAC Reactivity. J. Org. Chem. 2016, 81, 8850–8857. 10.1021/acs.joc.6b01545. PMC5999039 27635662

[ref19] Xing L.; Wang B.; Li J.; Guo X.; Lu X.; Chen X.; Sun H.; Sun Z.; Luo X.; Qi S.; Qian X.; Yang Y. A Fluorogenic ONOO(−)-Triggered Carbon Monoxide Donor for Mitigating Brain Ischemic Damage. J. Am. Chem. Soc. 2022, 144, 2114–2119. 10.1021/jacs.2c00094. 35080381

[ref20] Wang D.; Viennois E.; Ji K.; Damera K.; Draganov A.; Zheng Y.; Dai C.; Merlin D.; Wang B. A click-and-release approach to CO prodrugs. Chem. Commun. 2014, 50, 15890–3. 10.1039/C4CC07748B. 25376496

[ref21] Min Q.; Ni Z.; You M.; Liu M.; Zhou Z.; Ke H.; Ji X. Chemiexcitation-Triggered Prodrug Activation for Targeted Carbon Monoxide Delivery. Angew. Chem., Int. Ed. 2022, 61, e202200974 10.1002/anie.202200974. 35385195

[ref22] Zheng Y.; Ji X.; Yu B.; Ji K.; Gallo D.; Csizmadia E.; Zhu M.; Choudhury M. R.; De La Cruz L. K. C.; Chittavong V.; Pan Z.; Yuan Z.; Otterbein L. E.; Wang B. Enrichment-triggered prodrug activation demonstrated through mitochondria-targeted delivery of doxorubicin and carbon monoxide. Nat. Chem. 2018, 10, 787–794. 10.1038/s41557-018-0055-2. PMC6235738 29760413

[ref23] Kueh J. T. B.; Stanley N. J.; Hewitt R. J.; Woods L. M.; Larsen L.; Harrison J. C.; Rennison D.; Brimble M. A.; Sammut I. A.; Larsen D. S. Norborn-2-en-7-ones as physiologically-triggered carbon monoxide-releasing prodrugs. Chem. Sci. 2017, 8, 5454–5459. 10.1039/C7SC01647F. PMC5609517 28970925

[ref24] Steiger C.; Lühmann T.; Meinel L. Oral drug delivery of therapeutic gases - carbon monoxide release for gastrointestinal diseases. J. Controlled Release 2014, 189, 46–53. 10.1016/j.jconrel.2014.06.025. 24969354

[ref25] Steiger C.; Uchiyama K.; Takagi T.; Mizushima K.; Higashimura Y.; Gutmann M.; Hermann C.; Botov S.; Schmalz H. G.; Naito Y.; Meinel L. Prevention of colitis by controlled oral drug delivery of carbon monoxide. J. Controlled Release 2016, 239, 128–36. 10.1016/j.jconrel.2016.08.030. 27578097

[ref26] Southam H. M.; Williamson M. P.; Chapman J. A.; Lyon R. L.; Trevitt C. R.; Henderson P. J. F.; Poole R. K. ‘Carbon-Monoxide-Releasing Molecule-2 (CORM-2)’ Is a Misnomer: Ruthenium Toxicity, Not CO Release, Accounts for Its Antimicrobial Effects. Antioxidants (Basel). 2021, 10, 915 10.3390/antiox10060915. PMC8227206 34198746

[ref27] Yuan Z.; Yang X.; Ye Y.; Tripathi R.; Wang B. Chemical Reactivities of Two Widely Used Ruthenium-Based CO-Releasing Molecules with a Range of Biologically Important Reagents and Molecules. Anal. Chem. 2021, 93, 5317–5326. 10.1021/acs.analchem.1c00533. PMC8248650 33745269

[ref28] Yuan Z.; Yang X.; Wang B. Redox and catalase-like activities of four widely used carbon monoxide releasing molecules (CO-RMs). Chem. Sci. 2021, 12, 13013–13020. 10.1039/D1SC03832J. PMC8513939 34745532

[ref29] Ji X.; Aghoghovbia R. E.; De La Cruz L. K. C.; Pan Z.; Yang X.; Yu B.; Wang B. Click and Release: A High-Content Bioorthogonal Prodrug with Multiple Outputs. Org. Lett. 2019, 21, 3649–3652. 10.1021/acs.orglett.9b01086. 31063383

[ref30] Ji X.; Pan Z.; Li C.; Kang T.; De La Cruz L. K. C.; Yang L.; Yuan Z.; Ke B.; Wang B. Esterase-Sensitive and pH-Controlled Carbon Monoxide Prodrugs for Treating Systemic Inflammation. J. Med. Chem. 2019, 62, 3163–3168. 10.1021/acs.jmedchem.9b00073. 30816714

[ref31] Pan Z.; Zhang J.; Ji K.; Chittavong V.; Ji X.; Wang B. Organic CO Prodrugs Activated by Endogenous ROS. Org. Lett. 2018, 20, 8–11. 10.1021/acs.orglett.7b02775. 29111756

[ref32] Ji X.; Zhou C.; Ji K.; Aghoghovbia R. E.; Pan Z.; Chittavong V.; Ke B.; Wang B. Click and Release: A Chemical Strategy toward Developing Gasotransmitter Prodrugs by Using an Intramolecular Diels-Alder Reaction. Angew. Chem., Int. Ed. Engl. 2016, 55, 15846–15851. 10.1002/anie.201608732. 27879021

[ref33] Ji X.; Wang B. Strategies toward Organic Carbon Monoxide Prodrugs. Acc. Chem. Res. 2018, 51, 1377–1385. 10.1021/acs.accounts.8b00019. 29762011

[ref34] De La Cruz L. K.; Yang X.; Menshikh A.; Brewer M.; Lu W.; Wang M.; Wang S.; Ji X.; Cachuela A.; Yang H.; Gallo D.; Tan C.; Otterbein L.; de Caestecker M.; Wang B. Adapting decarbonylation chemistry for the development of prodrugs capable of in vivo delivery of carbon monoxide utilizing sweeteners as carrier molecules. Chem. Sci. 2021, 12, 10649–10654. 10.1039/D1SC02711E. PMC8356820 34447558

[ref35] Yang X.; Lu W.; Wang M.; De La Cruz L. K.; Tan C.; Wang B. Activated charcoal dispersion of carbon monoxide prodrugs for oral delivery of CO in a pill. Int. J. Pharm. 2022, 618, 121650 10.1016/j.ijpharm.2022.121650. PMC9060424 35276229

[ref36] Younes M.; Aggett P.; Aguilar F.; Crebelli R.; Dusemund B.; Filipič M.; Frutos M. J.; Galtier P.; Gott D.; Gundert-Remy U.; Kuhnle G. G.; Leblanc J. C.; Lillegaard I. T.; Moldeus P.; Mortensen A.; Oskarsson A.; Stankovic I.; Waalkens-Berendsen I.; Woutersen R. A.; Wright M.; Boon P.; Chrysafidis D.; Gürtler R.; Mosesso P.; Parent-Massin D.; Tobback P.; Kovalkovicova N.; Rincon A. M.; Tard A.; Lambré C. Re-evaluation of silicon dioxide (E 551) as a food additive. EFSA J. 2018, 16, e05088 10.2903/j.efsa.2018.5088. PMC7009582 32625658

[ref37] Diab R.; Canilho N.; Pavel I. A.; Haffner F. B.; Girardon M.; Pasc A. Silica-based systems for oral delivery of drugs, macromolecules and cells. Adv. Colloid Interface Sci. 2017, 249, 346–362. 10.1016/j.cis.2017.04.005. 28473052

[ref38] Zhou Y.; Yu W.; Cao J.; Gao H. Harnessing carbon monoxide-releasing platforms for cancer therapy. Biomaterials. 2020, 255, 120193 10.1016/j.biomaterials.2020.120193. 32569866

[ref39] Jaganathan H.; Godin B. Biocompatibility assessment of Si-based nano- and micro-particles. Adv. Drug Deliv Rev. 2012, 64, 1800–19. 10.1016/j.addr.2012.05.008. PMC3465530 22634160

[ref40] Reineke J. J.; Cho D. Y.; Dingle Y. T.; Morello A. P. 3rd; Jacob J.; Thanos C. G.; Mathiowitz E. Unique insights into the intestinal absorption, transit, and subsequent biodistribution of polymer-derived microspheres. Proc. Natl. Acad. Sci. U. S. A. 2013, 110, 13803–8. 10.1073/pnas.1305882110. PMC3752225 23922388

[ref41] Padmanabhan P.; Grosse J.; Asad A. B.; Radda G. K.; Golay X. Gastrointestinal transit measurements in mice with 99mTc-DTPA-labeled activated charcoal using NanoSPECT-CT. EJNMMI Res. 2013, 3, 60 10.1186/2191-219X-3-60. PMC3737085 23915679

[ref42] Zhu M.; Lerum M. Z.; Chen W. How to prepare reproducible, homogeneous, and hydrolytically stable aminosilane-derived layers on silica. Langmuir. 2012, 28, 416–23. 10.1021/la203638g. PMC3243110 22128807

[ref43] Zhi C.; Wang J.; Luo B.; Li X.; Cao X.; Pan Y.; Gu H. The synthesis of cyclohexenone using l-proline immobilized on a silica gel catalyst by a continuous-flow approach. RSC Advances. 2014, 4, 15036–15039. 10.1039/c4ra01231c.

[ref44] Han A.; Chen X.; Qiao Y. Effects of the Addition of Electrolyte on Liquid Infiltration in a Hydrophobic Nanoporous Silica Gel. Langmuir. 2008, 24, 7044–7047. 10.1021/la800446z. 18564859

[ref45] Wang M.; Yang X.; Pan Z.; Wang Y.; De La Cruz L. K.; Wang B.; Tan C. Towards ″CO in a pill″: Pharmacokinetic studies of carbon monoxide prodrugs in mice. J. Controlled Release 2020, 327, 174–185. 10.1016/j.jconrel.2020.07.040. PMC7606817 32745568

[ref46] Belyakova L. A.; Varvarin A. M. Surfaces properties of silica gels modified with hydrophobic groups. Colloids Surf. A: Physicochem Eng. Asp. 1999, 154, 285–294. 10.1016/S0927-7757(98)00650-5.

[ref47] Yang X.; Lu W.; Hopper C. P.; Ke B.; Wang B. Nature’s marvels endowed in gaseous molecules I: carbon monoxide and its physiological and therapeutic roles. Acta Pharm. Sin B 2021, 11, 1434–1445. 10.1016/j.apsb.2020.10.010. PMC8245769 34221861

[ref48] Zheng Y.; Yu B.; Ji K.; Pan Z.; Chittavong V.; Wang B. Esterase-Sensitive Prodrugs with Tunable Release Rates and Direct Generation of Hydrogen Sulfide. Angew. Chem., Int. Ed. Engl. 2016, 55, 4514–8. 10.1002/anie.201511244. PMC4902284 26822005

[ref49] Bakalarz D.; Surmiak M.; Yang X.; Wójcik D.; Korbut E.; Śliwowski Z.; Ginter G.; Buszewicz G.; Brzozowski T.; Cieszkowski J.; Głowacka U.; Magierowska K.; Pan Z.; Wang B.; Magierowski M. Organic carbon monoxide prodrug, BW-CO-111, in protection against chemically-induced gastric mucosal damage. Acta Pharm. Sin B 2021, 11, 456–475. 10.1016/j.apsb.2020.08.005. PMC7893125 33643824

