# Supplementary material for: Toward “CO in a Pill”: Silica-Immobilized Organic CO Prodrugs for Studying the Feasibility of Systemic Delivery of CO via In Situ Gastrointestinal CO Release
Source: Mol Pharm. 2023 Feb 20;20(3):1850–6. doi: 10.1021/acs.molpharmaceut.2c01104 (PMC9997063; doi:10.1021/acs.molpharmaceut.2c01104)
Supplement: Supplementary file 1 — mp2c01104_si_001.pdf [file mp2c01104_si_001.pdf]

Supplementary Information

Toward "CO in a pill:" Silica-immobilized Organic CO Prodrugs for Studying the Feasibility of Systemic Delivery of CO *via* in-situ Gastrointestinal CO Release

Xiaoxiao Yang[‡,a], *Ravi Tripathi*[‡,a], *Minjia Wang*[b], *Wen Lu*[a], *Abiodun Anifowose*[a], *Chalet Tan*[c], *and Binghe Wang*[a,*]

[‡] These authors contributed equally to this work

[a] Department of Chemistry and Center for Diagnostics and Therapeutics, Georgia State University, Atlanta, GA 30303, United States

[b] Department of Pharmaceutics and Drug Delivery, University of Mississippi School of Pharmacy, MS 38677, USA.

[c] Department of Pharmaceutical Sciences, University of Tennessee Health Science Center, Memphis, TN 38613, United States

*Corresponding author: B.W.: wang@gsu.edu; Phone: +1(404)-413-5544

<u>Table of contents</u>

1. Supplemental Figures and Tables……….…….………………….………………2-4
2. Material and Methods…………………………………………………….........5-9
3. Chemical Synthesis and Characterization of SICOs …………..……..………..…..…..9-13
4. NMR Spectra…………………………………………….…………………………14-17
5. References…………………………………………………………………………...18

Supplemental Figures

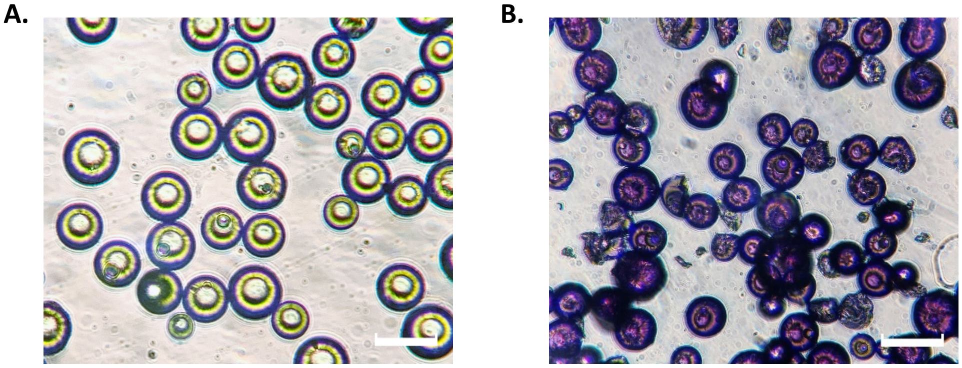

Figure S1. Spherical silica gels before (**A**) and after immobilization of CO prodrug (**B**) (scale bar: 50 μm).

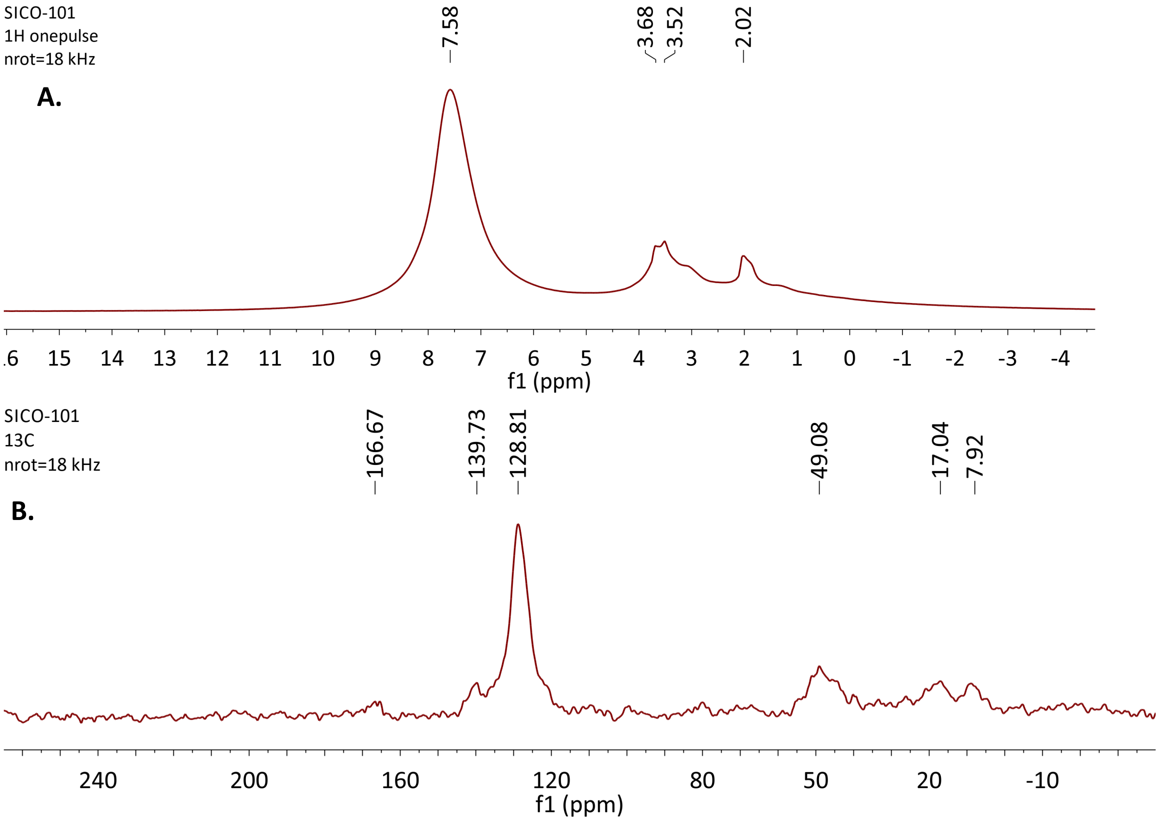

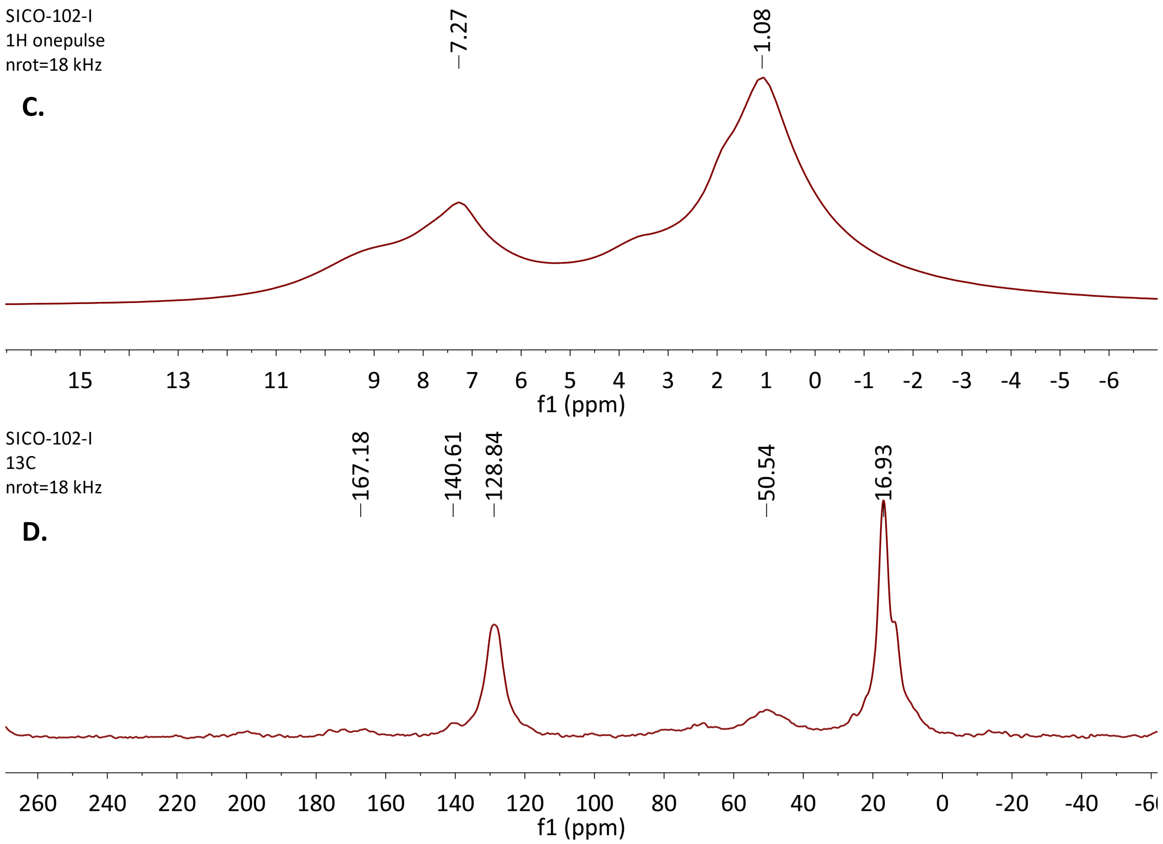

Figure S2. Magic-Angle-Spinning (MAS) NMR of **SICO-101** (**A**, ^{1}H-NMR; **B**, ^{13}C-NMR) and **SICO-102** (**C**, ^{1}H-NMR; **D**, ^{13}C-NMR).

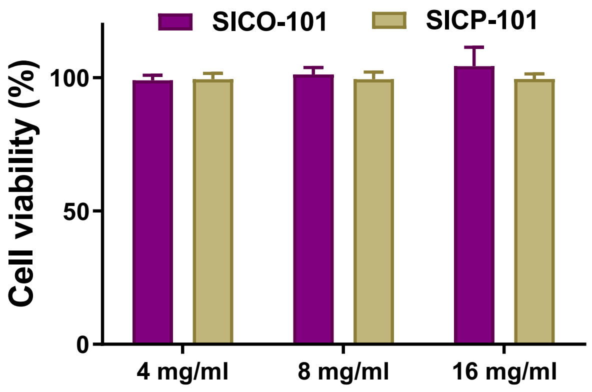

Figure S3. Cell viability tests (24 h) of RAW246.7 cells incubated with **SICO-101** and **SICP-101** with Transwell cell culture insert (mean ± SD, n = 3).

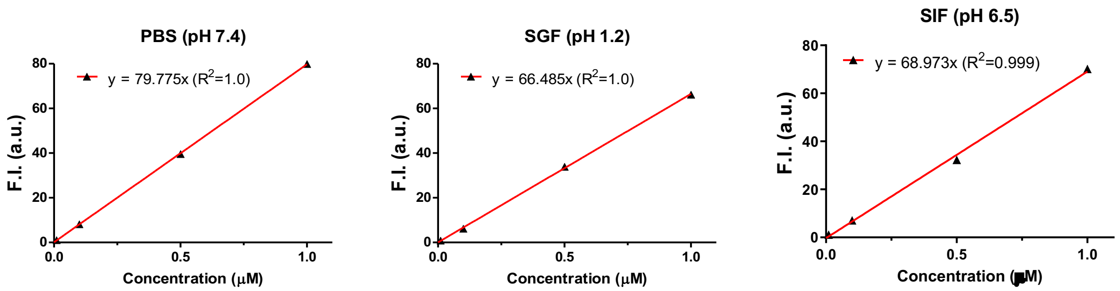

Figure S4. Standard curves of **SICP-102-D** in SGF, SIF, and PBS using fluorescence spectrophotometer. (**SICP-102-D** was used as a surrogate for the detached CO-released byproduct of **SICO-101** due to the similar fluorescence response and the technical difficulty in getting the standard compound for the detached **SICP-101**).

Table S1.

Medium	Fluorescence intensity (a.u.) in the supernatant	Calculated concentration of SICP-101 (nM) in the supernatant	Fraction of SICO-101 used in the experiment (ppm)
PBS	3.535	45.5	68.2
SGF	2.557	38.5	57.7
SIF	2.979	43.2	64.8

Table S2. Preliminary stability results of **SICO-101** after storage for 1-month at different temperature.

Storage temperature	-20 ℃	4 ℃	25 ℃
CO release yield*	94.1%	74.6%	4.8%

* CO release yield of freshly made **SICO-101** was designated as 100%.

Material and Methods

Gas chromatography studies

Gas chromatography was analyzed on an Agilent 7820A GC system by using either a TCD detector or a methanizer-FID detector. When a TCD detector is used, Agilent 7820A system is equipped with a thermal conductivity detector (TCD) and a Supelco Carboxen 1000 carbon molecular sieve column (15 ft × 1/8 in × 2.1 mm) using argon as the carrier gas. GC conditions: total flow: 30 mL/min; reference flow: 30 mL/min, TCD detector: 125 °C; oven temperature program: 35 °C (0-5 min), 35-225 °C (ramp: 20 °C/min), 225 °C (hold 5 min), 225-35 °C (ramp: 50 °C/min). When a methanizer-FID detector is used, Agilent 7820A system is equipped with purged packed inlet (operates at 150°C), Restek 5A mole sieve column (2m, 0.53mm ID, helium carrier gas at 4.5 mL/min flow rate), Restek CH4izer methanizer coupled with FID detector (methanizer H2 flow rate 25 mL/min, FID H2 flow rate 15 mL/min, air flow rate 400 mL/min, methanizer temperature 380 °C, FID temperature 300 °C), oven temperature program: 0-4 min 100 °C then increase to 250 °C at a rate of 60 °C/min and hold at 250 °C for 4 min followed by decrease to 100 °C at a rate of 60 °C/min then hold at 100 °C for 2 min. Cross-Polarization Magic-Angle-Spinning (MAS) solid-state NMR data were acquired on a Bruker Avance III HD 500 NMR spectrometer at the Georgia Institute of Technology.

Determination of CO release profiles using gas chromatography (TCD detector method)

An Agilent 7820A GC System equipped with a thermal conductivity detector (TCD) was used to detect and quantify CO release yield of the CO prodrugs. To make the standard curve, various volumes (25-500 μL) of pure CO gas were injected into the 6 mL headspace vials (8.8 mL total volume) containing 3 mL water. Using a gas-tight syringe, 250 μL of the headspace gas (5.8 mL) was drawn and injected into the injector port (purged packed inlet) maintained at 125 °C. Helium was used as the carrier gas with a flow rate of 30 mL/min. Gaseous components of the headspace were separated by passing through a packed column with 60/80 Carboxen-1000 matrix support, L × O.D. × I.D. 15.0 ft (4.6 m) × 1/8 in. × 2.1 mm (Supelco). The column was heated at 35 °C for 5 min, then 225 °C at 20 °C/min while the TCD detector was held at 125 °C. Under these conditions, CO had an elution time of around 7.4 min, while CO_2 eluted at 13.4 min. The peak area of CO was plotted against CO concentrations in the headspace to generate the standard curve for CO determination.

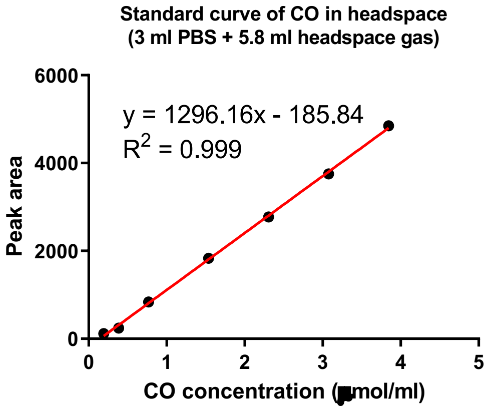

To test the CO release yield, about 20-50 mg **SICO**s were accurately weighed into a 6 mL headspace vial (8.8 mL actual total volume). Then 3 mL PBS was added into the headspace vial, and the vial was instantly sealed with a crimp cap with PTFE membraned silicone rubber septum and incubated overnight at 65 °C in an oven. 250 μL headspace gas was injected into the GC, and CO concentration was determined with the standard curve shown above.

To test the CO release kinetic, about 20-50 mg **SICO**s were precisely weighed into a 6 mL headspace vial (8.8 mL total volume). Then 3 mL prewarmed (37 °C) medium (PBS or SGF) was added into the headspace vial, and the vial was instantly sealed with a crimp cap with PTFE membraned silicone rubber septum and incubated at 37 °C in the incubator. At designated time points, 250 μL headspace gas was injected into the GC, followed by reinjecting 250 μL ambient air back to the headspace vial to balance the pressure. CO concentration was determined with the standard curve shown above, and the lost CO due to each injection was compensated by calculation. Non-linear regression of the data points was applied by using GraphPad Prism 9.0 based on one-phase association equation (a variant of first-order reaction kinetic equation: $Y = Y_0 + (Y_{max} - Y_0) \times (1 - e^{-kx})$ to calculate the CO release half-life ($t_{1/2}$) based on the regression value for k ($t_{1/2}$ = ln2/k).

CO myoglobin assay

Direct detection of CO release was done through a "one-compartment" Mb-CO assay. The assay cuvette (3 mL) containing the deoxy-Mb solution was added with an excessive amount of **SICO-101** (about 10 mg). The deoxy-Mb solution was prepared by degassing a solution of myoglobin in PBS (1 mg/mL, pH = 7.4) with nitrogen for at least 30 min, and then converted to deoxy-Mb by adding a freshly prepared solution of sodium dithionite (1 mL, 22 mg/mL). The whole set-up was then incubated at 37 °C with continuous shaking agitation. At the end

of 1 h, the cuvette was allowed to stand still for 30 min to settle down the silica gel. The clear portion of the cuvette was analyzed with a UV-Vis spectrometer.

Storage stability evaluation

About 15 mg **SICO-101** was precisely weighed into a 6-ml headspace vial (8.8 mL actual total volume) and stored at different temperature for 1 month. PBS (3 mL) was added, and the vial was sealed with a crimp cap with PTFE membraned silicone rubber septum and incubated overnight at 65 °C in an oven. 100 μL headspace gas was injected into the GC, and CO concentration was determined with a methanizer-FID detector using the standard curve established by injecting 100 μL CO calibration gas (10, 20, 50, and 100 ppm, Gasco, Oldsmar, USA).

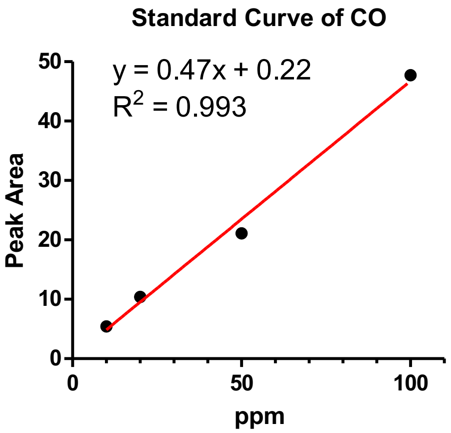

Determination of detached CO-released byproduct in the medium

5 mg **SICO-101** was mixed with 1.5 mL of either SGF or PBS in a vial. Next, the sealed vial was kept at 37 °C. After 24 h, the detection of the detached fluorescent CO-released byproduct (**SICP-102-D**) was conducted using a fluorescence spectrophotometer. The detached CO-released byproduct of **SICO-101** tends to tightly bind to the silica gel, thus making it impractical to purify. Since the substitution on the amide does not significantly change the fluorescence response of the CO-released compound, which was verified by comparing the same concentrations of **BW-CP-103** and **SICP-102-D**. **SICP-102-D** was used as the surrogate for the detached CO-released byproduct of **SICO-101**.

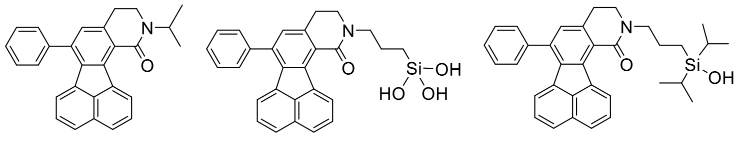

BW-CP-103 SICP-101-D SICP-102-D

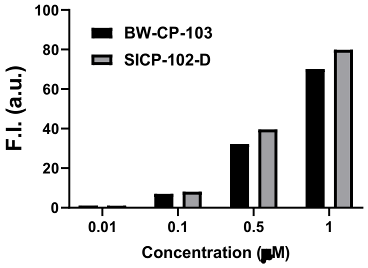

ELISA assay for TNF-α level in RAW264.7 cell culture

Anti-inflammatory effect of **SICO-101** was tested in RAW246.7 cells. Cells were cultured in the cell culture insert of the Transwell (6.5 mm diameter insert, 0.4 μm pore size) in the 24-well plate at a density of 5 × 10^4/well with DMEM medium supplemented with 10% fetus bovine serum and 1% penicillin and streptomycin. The upper compartment contains 100 μL culture medium, and the lower compartment contains 500 μL culture medium. After culturing overnight to allow cell adherence, **SICO-101** and **SICP-101** (see chemistry synthesis section for the procedure to make **SICP-101**) was weighed in 1.5 mL Eppendorf tubes and premixed with culture medium, then 500 μL of the slurry was transferred to the lower compartment of the 24-well plate to replace the existing culture medium. Then 6 μL of 0.1 μg/mL of LPS in PBS was added to the cell culture medium in the lower compartment and incubated for 24 h. The control group was added with 6 μL PBS. TNF-α concentration in the supernatant culture medium in the lower compartment was then determined with ELISA kit (Biolegend, USA) following the manufacturer's protocol.

Cytotoxicity assay in RAW264.7 cell culture

Cytotoxicity assay for **SICO-101** and **SICP-101** was tested in RAW246.7 cells. Cells were cultured in the cell culture insert of the Transwell (6.5 mm diameter insert, 0.4 μm pore size) in the 24-well plate at a density of 5 ×10^4/well with DMEM medium supplemented with 10% fetus bovine serum and 1% penicillin and streptomycin. The upper compartment contains 100 μL culture medium, and the lower compartment contains 500 μL culture medium. After culturing overnight to allow cell adherence, **SICO-101** and **SICP-101** were weighed in 1.5 mL Eppendorf tubes and premixed with culture medium; then 500 μL of the slurry was transferred to the lower compartment of the 24-well plate to replace the existing culture medium. The control group was incubated with 500 μL full culture medium without silica. After incubation

for 24 h, the cell culture inserts were transferred to a new 24-well plates containing 800 μL fresh culture medium with 10% CCK-8 (Dojindo, Kumamoto, Japan) solution. After incubation for 3 h, the Transwell inserts were removed from the 24-well plate and the optical density (O.D.) at 490 nm was directly read by a plate reader (Perkin-Elmer Victor2, Waltham, USA). The cell viability was calculated as: Viability (%) = (O.D. of treatment group/O.D. of the control group) ×100%

Pharmacokinetic Studies

CD-1 mice (25–30 g) were purchased from Envigo (Indianapolis, USA) and were fed with food and drinking water *ad libitum*. Animals were maintained in accordance with the guidelines for the Care and Use of Laboratory Animals of the National Institute of Health. All the animal protocols were approved by the Institutional Animal Care and Use Committee of the University of Mississippi (IACUC protocol: 19-012).

SICO-101 was mixed with 3% CMC in a 1:6 ratio (1 mg : 6 μL). The drug-loaded formulation or the blank vehicle was administered to fed mice (n ≥ 3) *via* oral gavage (*p.o.*) administration. At pre-determined time points post-administration, blood samples were collected by retro-orbital bleeding. Circulating CO was monitored by measuring the carboxyhemoglobin (COHb) level in the whole blood using a CO-oximeter (AVOXimeter 4000, Avox Systems, New York, NY, USA). The CO-oximetry measurements were made following the manufacturer's protocol and were validated by performing the specified quality control protocol. The baseline COHb level of each mouse was measured before administration. The area under the COHb level curve (COHb *AUC*) was estimated using GraphPad Prism (San Diego, CA, USA)

Chemical Synthesis and Characterization of SICOs

General information

Reagents were purchased from Sigma-Aldrich (Saint Louis, USA), Gelest, Inc. (USA), or Oakwood Chemical (USA). Moisture-sensitive reactions were carried out using anhydrous solvents purified by a Vigor Tech purification system (Houston, Texas, USA). Silica gel for the flash column was purchased from Sigma-Aldrich (Saint Louis, Missouri, USA). ^{1}H- (400 MHz) and ^{13}C-NMR (101 MHz) spectra were recorded on a Bruker Avance 400 MHz NMR spectrometer. Deuterated solvents were purchased from Cambridge Isotope Laboratories, Inc

(USA). Chemical shifts were reported as δ values (ppm). TMS (δ= 0 ppm) or residual peaks of the deuterated solvent were used as the internal reference. Mass spectrometry analyses were conducted by the Georgia State University Mass Spectrometry Facilities, which are partially supported by an NIH grant for the purchase of a Waters Xevo G2-XS Mass Spectrometer (1S10OD026764-01). High-resolution mass spectrometry (HRMS) analyses were conducted by the Georgia State University mass spectrometry facilities using ABI API 3200 (ESI-Triple Quadruple) instrument.

General Procedure for the Synthesis of Compound 1

3-(Triethoxysilyl)propylamine (for synthesis **1a**) or 3-(ethoxydiisopropylsilyl)propan-1-amine (7.5 mmol, 1.5 eq.) and triethylamine (6 mmol, 1.2 eq.) were stirred together in 15 mL anhydrous ACN in a 100 mL round-bottom flask. Next, 4-bromobut-1-yne (5 mmol, 1 eq.) diluted in ACN (3 mL) was added to the reaction mixture at 0.05 mL/min rate using a syringe pump. Further, the reaction was refluxed under an argon atmosphere and monitored by TLC (DCM: MeOH = 10:1). After completion of the reaction, the solvent was removed *in-vacuo* without further purification (for **1a**) or directly purified by silica-gel chromatography (for **1b**) to yield the desired product as pale-yellowish oil.

General Procedure for the Synthesis of Compound 3

Compound **1** (1.74 mmol) and 2,2-dimethyl-5-(2-phenylacetyl)-1,3-dioxane-4,6-dione (1.45 mmol, compound **2**, synthesized according to a reported procedure[1]) were dissolved, and stirred at 90 °C in dry toluene (8 mL) under an argon atmosphere. Next, trimethylsilyl chloride (2.17 mmol) was injected into the reaction mixture, and the reaction was allowed to reflux and monitored by TLC (hexane: EtOAc = 2:1) till the consumption of the starting material **2**. Then, the reaction mixture was diluted with EtOAc (15 mL), washed with water (3 × 10 mL), brine (3 × 10 mL), and dried over Na_2SO_4. Further, the solvent was concentrated completely under reduced pressure and purified by silica gel column chromatography to afford desired product **3a** or **3b** as light yellowish oil.

Activation of spherical silica gel by the piranha solution

Spherical silica gels were cleaned and activated by the piranha solution (concentrated H_2SO_4:30% H_2O_2 (7:3)) applying the reported procedure.[2] Briefly, 10 g silica gel was mixed in a freshly prepared 100 mL piranha solution and stirred at ice-bath temperature. After 30 min, the

mixture was diluted with purified water (100 mL), filtered, and washed generously with purified water. Further, the activated silica gels were dried at 180 °C overnight in an oven.

General Procedure for the Synthesis of Compound 4

A dry 20 mL sealed reaction tube was charged with 200 mg activated silica gel, followed by the addition of compound **3** (0.18 mmol) dissolved in 1.5 mL dry DMF. A micro stirring bar was added to the reaction and stirred at the lowest speed (100 rpm) to avoid crashing the silica gel particles. The reaction was heated at 90 °C for 36 h, at which time the silica gel became yellowish. The mixture was filtered under reduced pressure, and the silica gel was soaked (5 min) and washed with methanol (10 mL × 5 times), DMF (10 mL × 5 times), and DCM (10 mL × 5 times). Then the silica gel was dried under reduced pressure.

General Procedure for the Synthesis of SICO-101 and SICO-102

In a 20 mL scintillation vial equipped with a micro stirring bar was added 200 mg silica gel, compound **4**, acenaphthoquinone (33 mg, 0.18 mmol), Et_3N (50 μL, 0.36 mmol), and 5 mL DMF. The reaction was stirred at 200 rpm at 45 °C for 1.5 h, at which point the silica gel became purplish brown. Sampling a few silica gel beads showed greenish fluorescence under 365 nm UV light, indicating the formation of the aldol condensation intermediate. Then the reaction was suction-filtered and soak-washed with DMF (10 mL × 5 times) and DCM (10 mL × 5 times) to remove unreacted acenaphthoquinone. The silica gel was then briefly dried under reduced pressure and transferred to a 20 mL scintillation vial containing 5 mL acetic anhydride. The mixture was then cooled to 0 °C followed by the addition of 2-3 drops of concentrated sulfuric acid and allowed to react under 500 rpm stirring for 30 min. Upon addition of the concentrated sulfuric acid, the silica gel gradually became purplish black. The black silica gel was suction filtered and successively washed with DMF (10 mL × 5), DCM, and hexanes (10 mL × 5) followed by drying overnight within a lyophilizer (100 pa, -70 °C cold trap). The target **SICOs** were stored in a -80 °C freezer before being tested for the further studies.

Procedure for the Synthesis of SICP-101

About 100 mg **SICO-101** was added in a 20 mL scintillation vial, followed by the addition of 10 mL ultra-pure water. The mixture was incubated at 100 °C in an oven until the color of the silica gel changed from purple to light grayish yellow (about 4-5 h). The supernatant was

decanted, and the residual silica gel was dried at 120 °C in an oven. This dried silica gel powder was used as **SICP-101** without further processing.

Procedure for the Synthesis of SICP-102-D

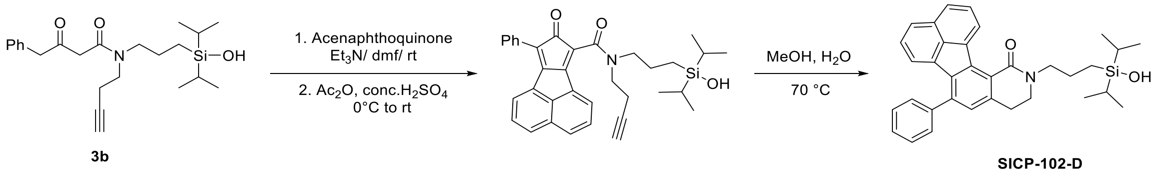

Compound **3b** (0.326 mmol), acenaphthaquinone (0.358 mmol), and Et_3N (0.489 mmol) were dissolved together in dry DMF (6 mL). The reaction was monitored by TLC (DCM: MeOH= 30:1) till the consumption of the starting material, and later the solvent was completely removed under reduced pressure. The reaction mixture was resuspended in acetic anhydride (10 mL) and stirred at ice-bath temperature. Next, 2-3 drops of concentrated H_2SO_4 were added to the solution, and the reaction was stirred for another 10 min. After completion of the reaction, the mixture was diluted with EtOAc (5 mL) and quenched by adding 10 mL water. The aqueous phase was extracted with EtOAc (3 × 10 mL). The combined organic phase was washed with saturated $NaHCO_3$ (3 x 10 mL), brine (3 × 10 mL) and dried over anhydrous Na_2SO_4. Further, the solvent was removed *in-vacuo,* and the residue was resuspended in 10 mL MeOH: H_2O (2:1). The reaction mixture was stirred at 50 °C till the completion of the reaction. After completion of the reaction, 30 mL water was added to the solution, and the crude product was extracted in EtOAc (20 mL) and further washed by water and brine (3 × 10 mL, each). Further, the organic layer was dried over anhydrous Na_2SO_4, concentrated *in-vacuo,* and the residue was purified by silica-gel chromatography to yield an off-white product.

(3-(but-3-yn-1-ylamino)propyl)diisopropylsilanol (1b):

Isolated yield: 10%. ^{1}H-NMR ($CDCl_3$) δ 7.18 (s, 1H), 3.67 (q, *J* = 6.9 Hz, 2H), 2.98 (t, *J* = 7.2 Hz, 2H), 2.89 – 2.81 (m, 2H), 2.68 (td, *J* = 7.2, 2.6 Hz, 2H), 2.03 (t, *J* = 2.6 Hz, 1H), 1.85 – 1.73 (m, 2H), 1.14 (t, *J* = 6.9 Hz, 3H), 0.97 (d, *J* = 1.8 Hz, 14H), 0.65 – 0.56 (m, 2H). ^{13}C-NMR ($CDCl_3$) δ 80.0, 71.0, 59.0, 51.3, 46.4, 21.4, 18.7, 17.7, 17.6, 17.3, 12.4, 7.8. Calculated for $C_{15}H_{31}NOSi$ $[M+H]^+$ 270.2253; Found 270.2243.

N-(but-3-yn-1-yl)-3-oxo-4-phenyl-*N*-(3-(triethoxysilyl)propyl)butanamide (3a):

Isolated yield: 19%. As reported in the previous publications,[1, 3] the compound exhibits tautomerism which results in complicated ^{1}H/^{13}C-NMR spectra. ^{1}H-NMR ($CDCl_3$) δ 14.81 (d, *J*

= 42.5 Hz, 1H), 7.35 – 7.12 (m, 8H), 5.02 (d, *J* = 6.7 Hz, 1H), 3.79 (ddd, *J* = 19.9, 10.4, 5.8 Hz, 10H), 3.57 (s, 1H), 3.45 (dd, *J* = 14.9, 7.3 Hz, 4H), 3.29 (dt, *J* = 14.1, 8.5 Hz, 2H), 3.24 – 3.10 (m, 2H), 2.43 (qd, *J* = 6.8, 2.6 Hz, 2H), 2.32 (ddd, *J* = 10.4, 9.4, 4.7 Hz, 1H), 1.96 (dt, *J* = 7.2, 2.5 Hz, 1H), 1.68 – 1.51 (m, 3H), 1.26 – 1.13 (m, 13H), 0.61 – 0.52 (m, 1H), 0.52 – 0.41 (m, 2H). ^{13}C-NMR ($CDCl_3$) δ 202.3, 202.0, 176.7, 176.4, 171.8, 171.6, 166.7, 166.6, 166.5, 136.3, 133.7, 129.6, 129.1, 128.7, 128.7, 128.4, 127.1, 126.8, 87.6, 87.4, 81.7, 81.6, 80.3, 71.1, 70.6, 69.7, 58.4, 58.4, 51.6, 51.2, 49.9, 49.8, 48.6, 48.1, 48.0, 47.6, 46.8, 46.6, 45.4, 45.1, 42.2, 22.3, 20.8, 18.6, 18.3, 18.3, 17.9, 17.4, 7.5, 7.5, 7.4, 7.4, 7.3. Calculated for $C_{23}H_{35}NO_5Si$ $[M-H]^-$ 432.2206; Found 432.2199. ^{1}H-NMR integration indicates the ratio of the tautomers being 1:1.72.

N-(but-3-yn-1-yl)-N-(3-(hydroxydiisopropylsilyl)propyl)-3-oxo-4-phenylbutanamide (3b):

Isolated yield: 17%. As reported in the previous publications,[1, 3] the compound exhibits tautomerism, which results in complicated ^{1}H/^{13}C-NMR spectra. ^{1}H-NMR ($CDCl_3$) δ 7.37 – 7.18 (m, 10H), 5.00 (d, *J* = 13.9 Hz, 1H), 3.85 (d, *J* = 1.7 Hz, 3H), 3.61 (s, 1H), 3.55 – 3.41 (m, 5H), 3.38 – 3.25 (m, 4H), 3.22 – 3.11 (m, 2H), 2.50 – 2.41 (m, 2H), 2.40 – 2.28 (m, 2H), 1.98 (dd, *J* = 5.6, 2.7 Hz, 2H), 1.73 – 1.62 (m, 2H), 1.55 (ddd, *J* = 16.3, 10.1, 6.6 Hz, 2H), 1.06 – 0.88 (m, 28H), 0.54 (dd, *J* = 16.9, 9.0 Hz, 2H), 0.44 (dd, *J* = 15.8, 7.3 Hz, 2H). ^{13}C-NMR ($CDCl_3$) δ 202.4, 202.3, 176.8, 176.7, 171.8, 171.6, 167.1 166.7, 136.3, 133.6, 133.6, 129.7, 129.6, 129.3, 129.2, 128.8, 128.8, 128.5, 127.3, 127.2, 126.9, 126.8, 87.7, 87.5, 81.9, 81.7, 80.4, 80.3, 71.2, 70.8, 69.8, 52.5, 52.1, 50.1, 49.8, 48.8, 48.4, 47.9, 46.6, 46.6, 45.5, 45.2, 42.2, 22.8, 21.7, 21.3, 18.8, 18.6, 18.0, 17.4, 17.4, 17.4, 13.0, 12.9, 8.3, 8.1, 7.7, 7.5. Calculated for $C_{23}H_{35}NO_3Si$ $[M+Na]^+$ 424.2284; Found 424.2276. ^{1}H-NMR integration indicates the ratio of the tautomers being 1:1.14.

2-(3-(hydroxydiisopropylsilyl)propyl)-6-phenyl-3,4-dihydroacenaphtho[1,2-*h*]isoquinolin-1(2*H*)-one (SICP-102-D):

Isolated yield: 76%. ^{1}H-NMR ($CDCl_3$) δ 9–.23 (d, *J* = 7.3 Hz, 1H), 7.86 (d, *J* = 8.1 Hz, 1H), 7.77 (d, *J* = 8.1 Hz, 1H), 7.68 (t, *J* = 7.7 Hz, 1H), 7.59 – 7.48 (m, 5H), 7.30 (t, *J* = 7.6 Hz, 1H), 7.06 (s, 1H), 7.02 (d, *J* = 7.1 Hz, 1H), 3.77 (t, *J* = 6.9 Hz, 2H), 3.62 (t, *J* = 6.2 Hz, 2H), 3.08 (t, *J* = 6.2 Hz, 2H), 1.91 (p, *J* = 7.3 Hz, 2H), 1.10 – 0.98 (m, 14H), 0.74 (t, *J* = 7.6 Hz, 2H). ^{13}C-NMR ($CDCl_3$) δ 165.0, 141.3, 140.6, 139.8, 138.5, 137.5, 135.3, 133.3, 129.7, 129.5, 129.0, 128.8, 128.5, 128.2, 127.9, 127.9, 127.0, 126.9, 125.6, 122.7, 49.9, 46.0, 30.4, 29.8, 22.1, 17.7, 17.6, 13.3, 7.8. Calculated for $C_{34}H_{37}NO_2Si$ $[M-H]^-$ 518.2515; Found 518.2511.

NMR Spectrum

Compound **1b**:

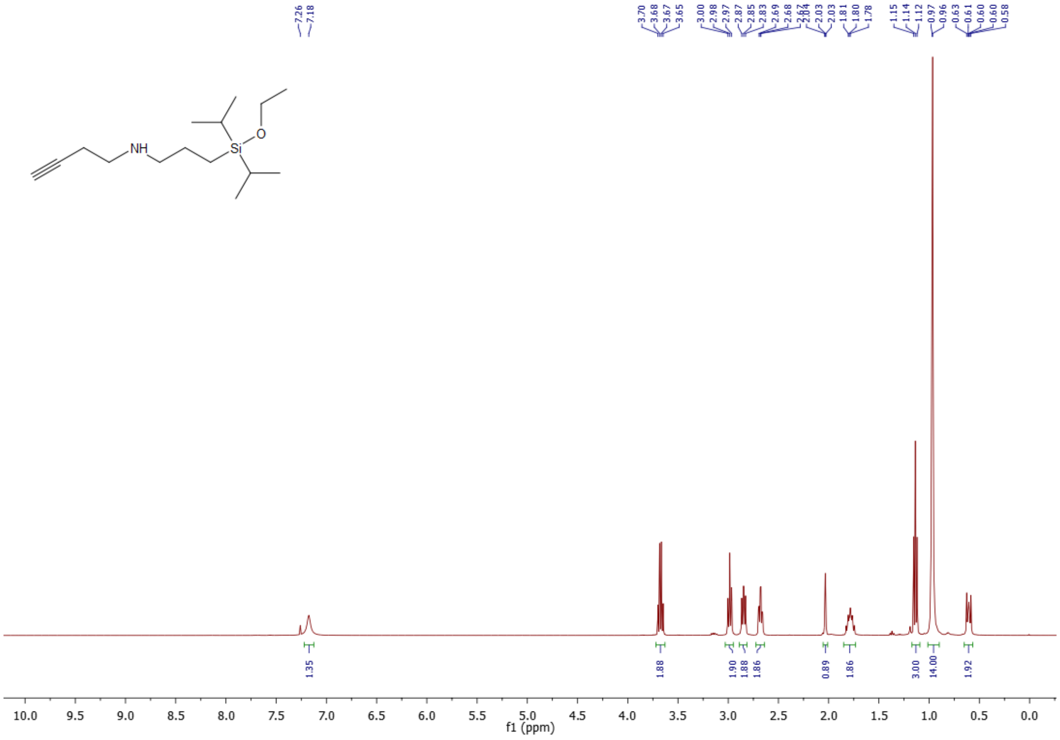

Compound **1b**:

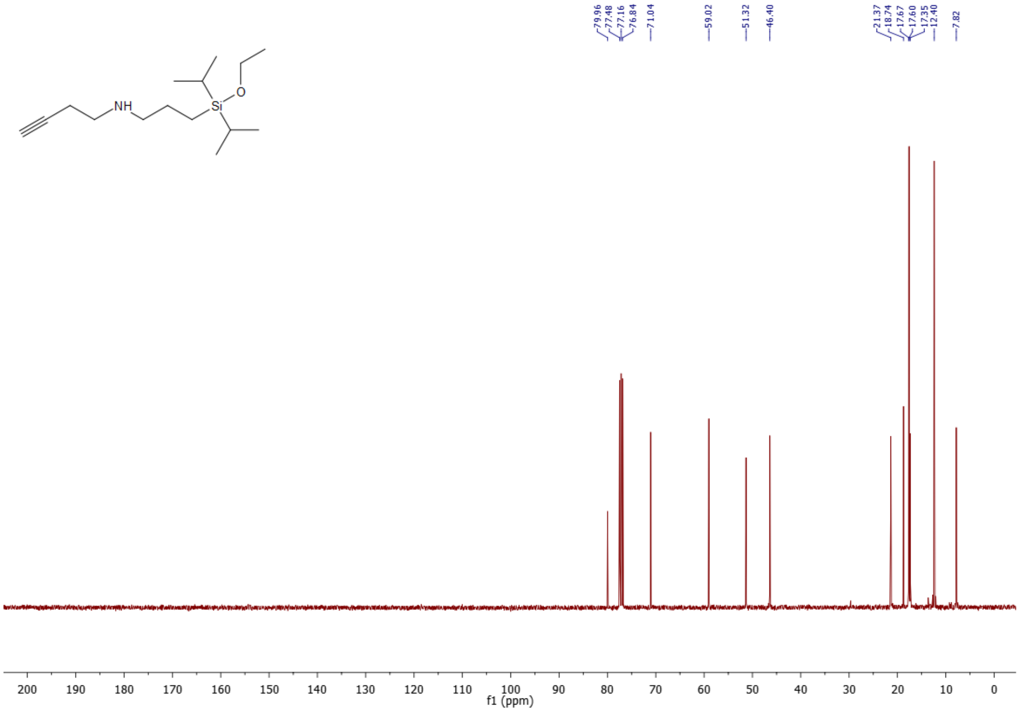

Compound **3a**:

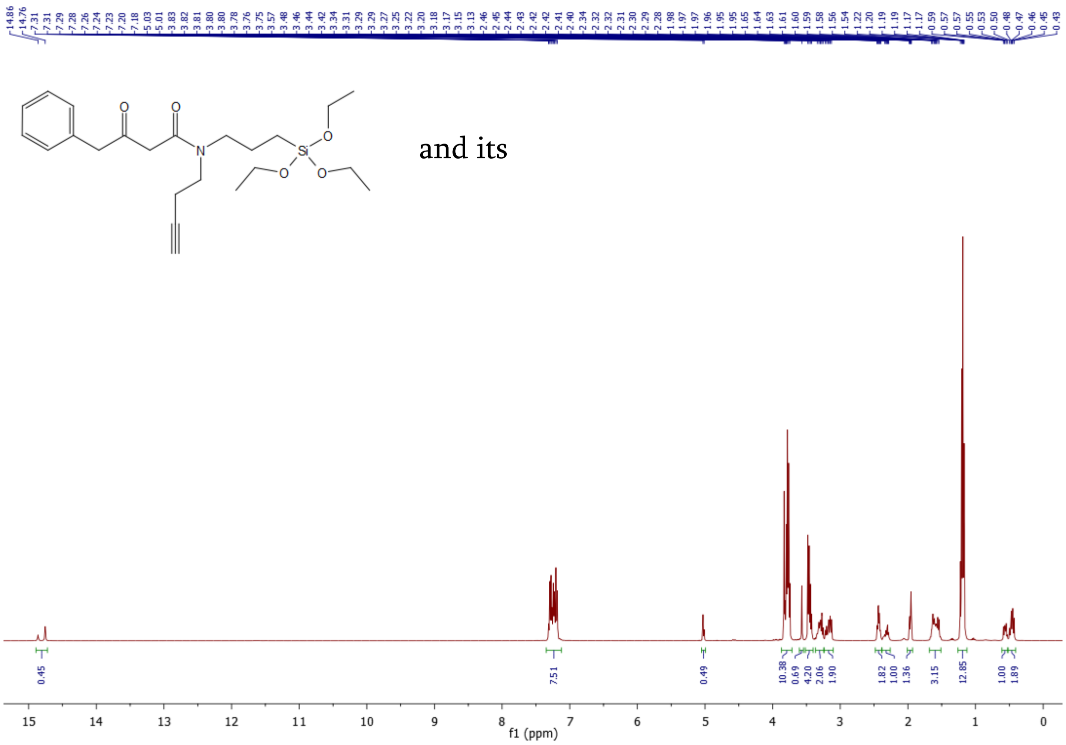

and its

Compound **3a**:

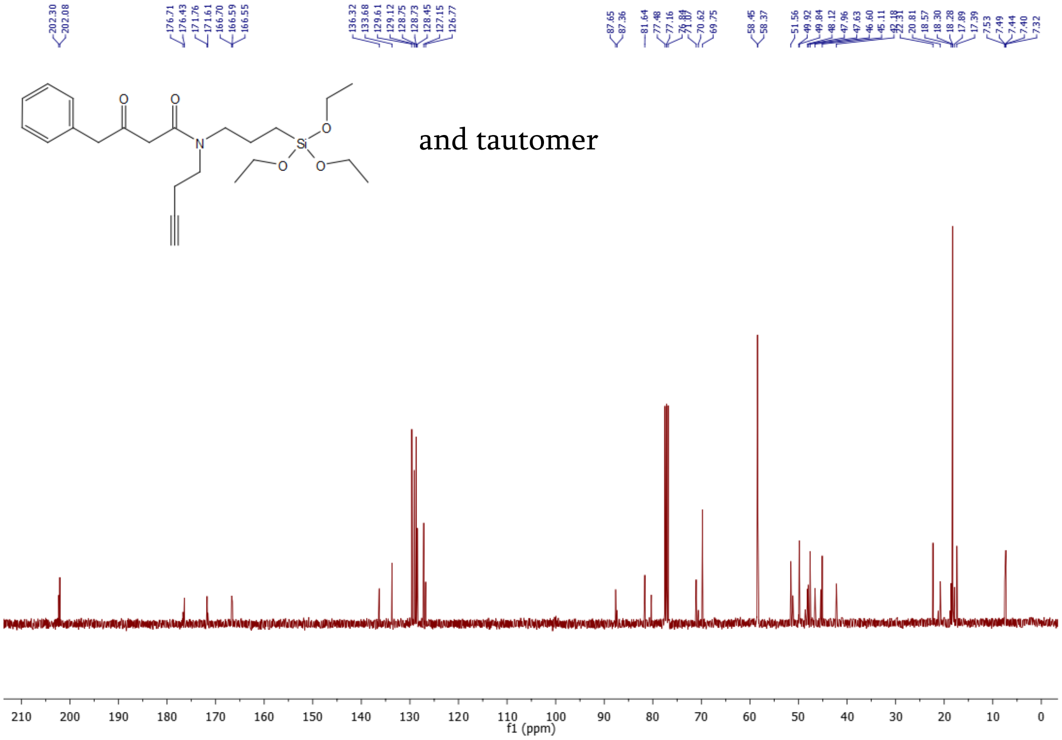

and tautomer

Compound **3b**:

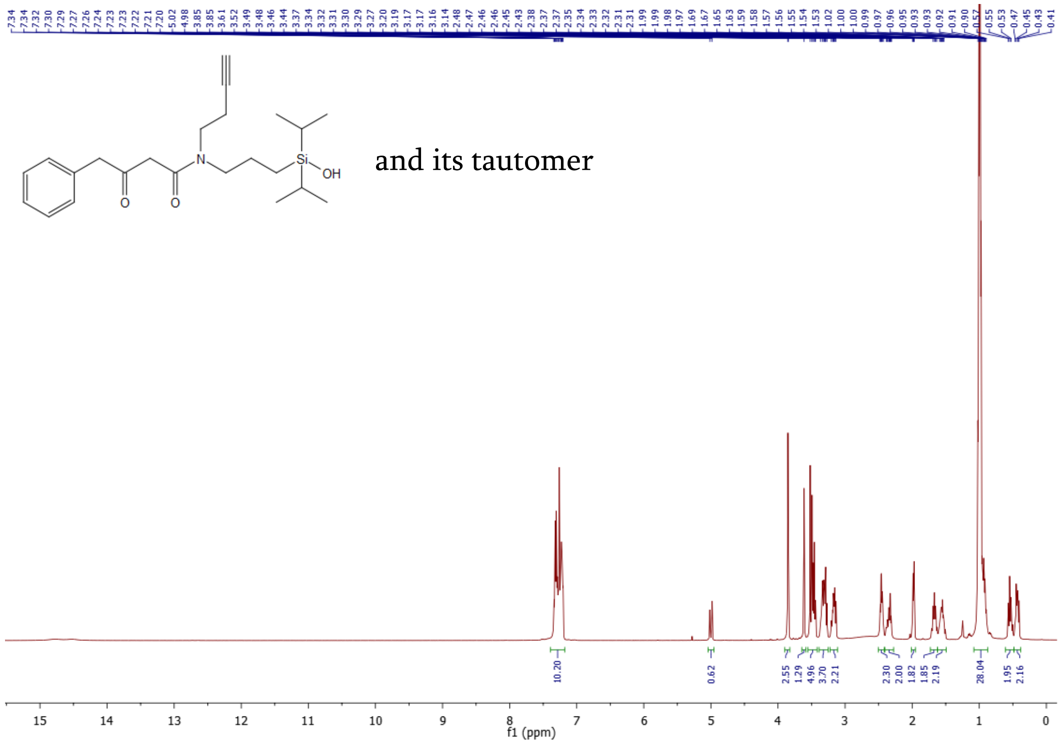

Compound **3b**:

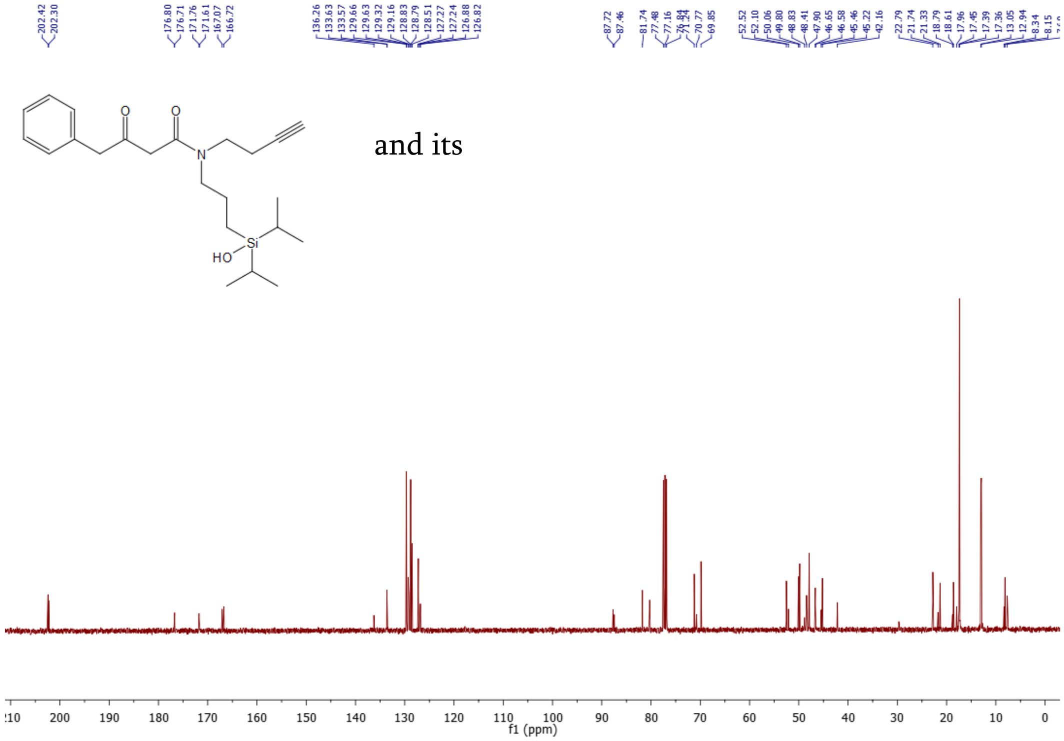

SICP-102-D:

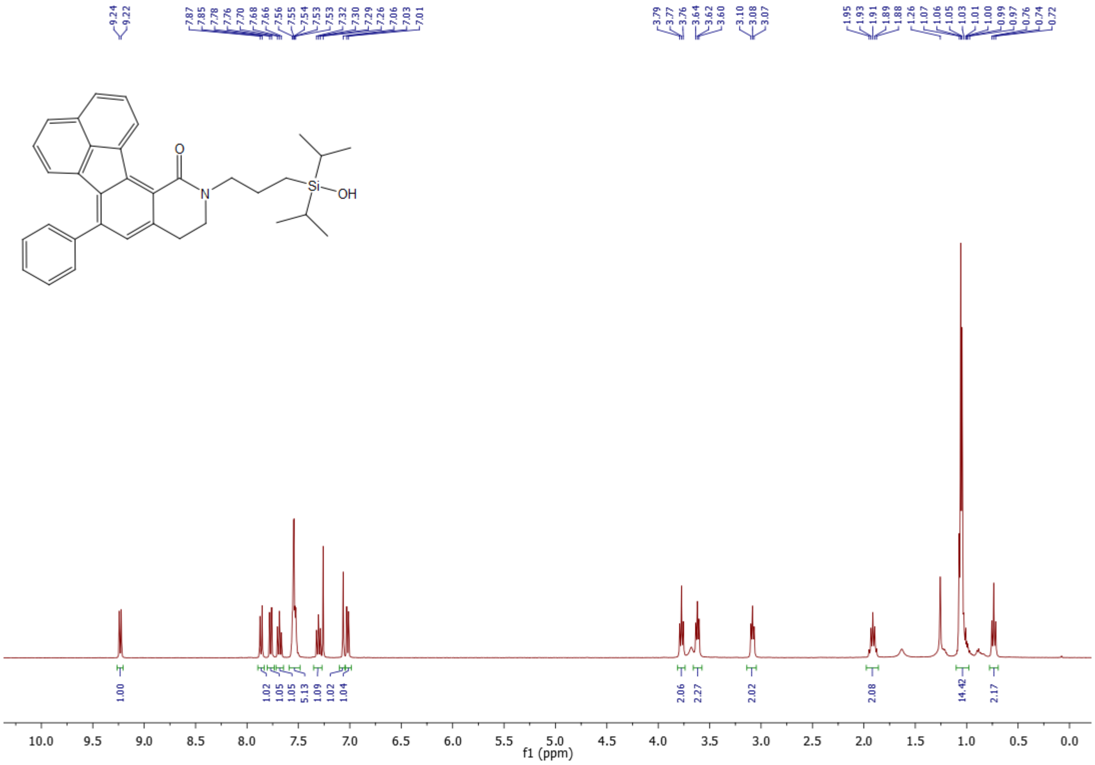

SICP-102-D:

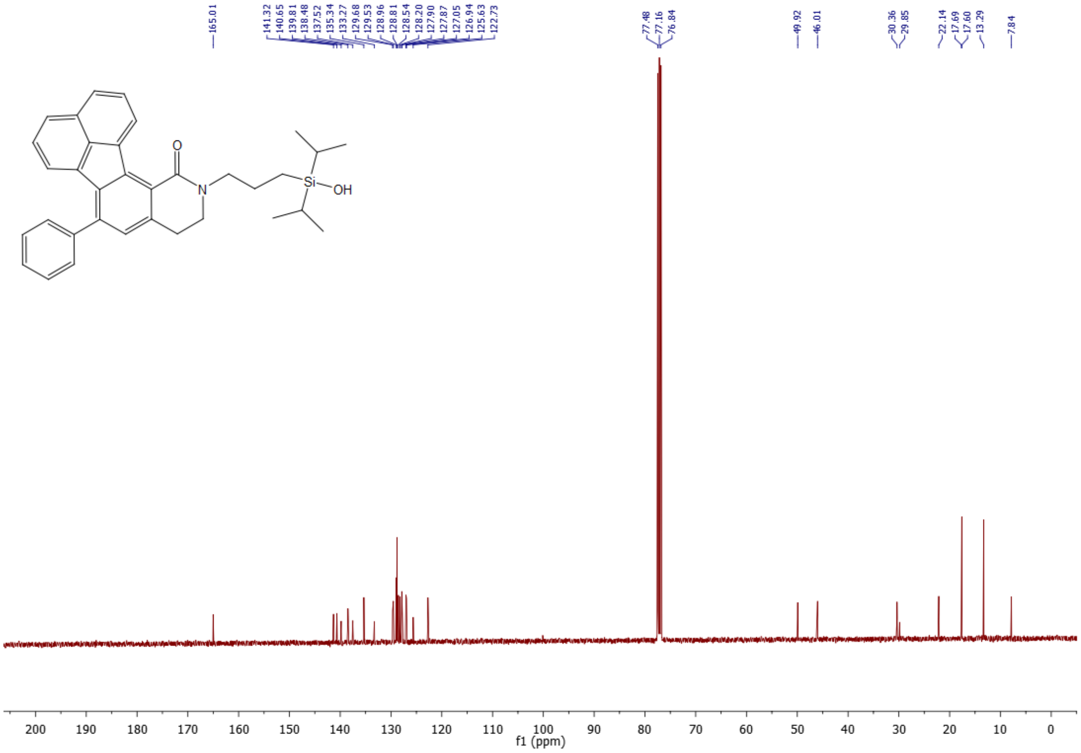

References:

1. Ji, X.; Zhou, C.; Ji, K.; Aghoghovbia, R. E.; Pan, Z.; Chittavong, V.; Ke, B.; Wang, B., Click and Release: A Chemical Strategy toward Developing Gasotransmitter Prodrugs by Using an Intramolecular Diels-Alder Reaction. *Angew Chem Int Ed Engl* **2016,** *55* (51), 15846-15851.
2. O'Mahony, T. F.; Morris, M. A., Hydroxylation methods for mesoporous silica and their impact on surface functionalisation. *Microporous Mesoporous Mater* **2021,** *317*, 110989.
3. Pan, Z.; Chittavong, V.; Li, W.; Zhang, J.; Ji, K.; Zhu, M.; Ji, X.; Wang, B., Organic CO Prodrugs: Structure-CO-Release Rate Relationship Studies. *Chemistry* **2017,** *23* (41), 9838-9845.
